# Multifunctional Solar Evaporator with Adjustable Island Structure Improves Performance and Salt Discharge Capacity of Desalination

**DOI:** 10.1002/advs.202305523

**Published:** 2023-10-24

**Authors:** Jianfei Wu, Ziwei Cui, Yang Yu, Bo Yue, Jundie Hu, Jiafu Qu, Jianzhang Li, Dan Tian, Yahui Cai

**Affiliations:** ^1^ Co‐Innovation Center of Efficient Processing and Utilization of Forest Resources, College of Materials Science and Engineering Nanjing Forestry University Nanjing 210037 P. R. China; ^2^ School of Chemical and Pharmaceutical Engineering Qilu University of Technology (Shandong Academy of Sciences) Jinan 250353 P. R. China; ^3^ School of Materials Science and Engineering Suzhou University of Science and Technology Suzhou 215009 P. R. China; ^4^ Key Laboratory of Wood Material Science and Application (Beijing Forestry University) Ministry of Education Beijing 100083 P. R. China; ^5^ Dehua Tubaobao New Decoration Material Co., Ltd Huzhou 313200 P. R. China

**Keywords:** adjustable structures, multifunctional evaporators, photo‐thermal‐electric devices, salt resistance, thermal management

## Abstract

Interfacial solar steam generation (ISSG) is the main method to get fresh water from seawater or wastewater. The balance between evaporation rate and salt resistance is still a major challenge for ISSG. Herein, a wood aerogel island solar evaporator (WAISE) with tunable surface structure and wettability by synthesizing poly(n‐isopropylacrylamide)‐modified multi‐walled carbon nanotube photothermal layers. Compared to dense surface structure evaporators, interfacial moisture transport, thermal localization, and surface water vapor diffusion of WAISE are greatly promoted, and the evaporation rate of WAISE increased by 87.64%. WAISE allows for record performance of 200 h continuous operation in 20% NaCl solution without salt accumulation. In addition, the photo‐thermal‐electric device is developed based on WAISE with continuous water purification, power generation, and irrigation functions. This work provides a new direction for the development of multifunctional water purification systems.

## Graphical abstract

In this paper, a wood aerogel island solar evaporator (WAISE) with tunable surface structure and wettability is prepared by synthesizing a poly(n‐isopropylacrylamide)‐modified multi‐walled carbon nanotubes photothermal material. WAISE has effectively balanced the contradiction between evaporation rate and salt tolerance, so that this work provides a new research idea for the application of a wood evaporator in seawater desalination.

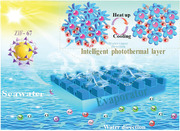

## 1 Introduction

These global freshwater crisis makes extracting fresh water from the abundant seawater a compelling solution.^[^ ^1^, ^2^ ^]^ Interfacial solar steam generation (ISSG) is a promising desalination technology for obtaining clean water from seawater^[^ ^3^, ^4^ ^]^ and wastewater.^[^ ^5^, ^6^, ^7^ ^]^ ISSG has advantages such as low cost,^[^ ^8^, ^9^, ^10^ ^]^ high efficiency,^[^ ^11^, ^12^, ^13^, ^14^, ^15^, ^16^ ^]^ and zero carbon emissions^[^ ^17^, ^18^, ^19^, ^20^ ^]^ compared to traditional desalination technologies (such as multi‐stage flash distillation).^[^ ^21^ ^]^

The factors such as photothermal materials (PTMs),^[^ ^22^ ^]^ local thermal management,^[^ ^23^, ^24^, ^25^, ^26^ ^]^ salt resistant structure^[^ ^27^, ^28^, ^29^, ^30^, ^31^ ^]^ and water transport channels^[^ ^32^ ^]^ are all needed to be considered for designing high performance evaporators. The choice of PTM is crucial for developing high evaporation rate evaporators.^[^ ^33^, ^34^, ^35^ ^]^ Typical PTMs include plasma metals,^[^ ^36^, ^37^ ^]^ semiconductor materials,^[^ ^38^, ^39^ ^]^ carbonaceous materials,^[^ ^40^, ^41^, ^42^, ^43^ ^]^ and polymers.^[^ ^44^, ^45^ ^]^ The impact of heat loss on the design of high performance evaporators cannot be ignored.^[^ ^46^ ^]^ Low thermal conductivity materials used as insulation to reduce contact between materials and bulk water effectively decrease heat loss.^[^ ^47^, ^48^ ^]^ In addition, prolonged interfacial evaporation leads to the accumulation of salt crystals on the evaporator surface,^[^ ^49^ ^]^ resulting in heat radiation loss and obstructing the transmission path of the water supply to reduce evaporation rates. Designing hydrophilic surfaces and enhancing fluid convection are the most studied salt tolerance strategies.^[^ ^50^, ^51^ ^]^ However, significant heat loss occurs due to excessive water coverage by overly hydrophilic surfaces,^[^ ^52^ ^]^ which makes evaporation difficult. Therefore, it is still a major challenge to balance the contradictions of the high salt tolerance of ISSG with the high evaporation rate. In addition, many random cross‐linked evaporators have distorted channels and uneven pore size distribution. The strongly viscous forces created by these factors severely impede water vapor diffusion, thus limiting water evaporation.^[^ ^53^ ^]^

**Scheme 1:**
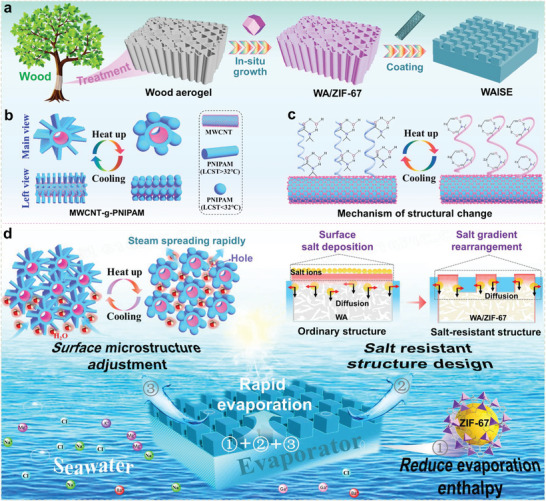
Preparation and application of WAISE. a) The preparation process of WAISE. b) Structural changes of MWCNT‐g‐PNIPAM in different views during heating and cooling. c) Structural change mechanism of MWCNT‐g‐PNIPAM. d) Salt‐tolerance mechanism of tunable surface structures and island structures in desalination process diagrams.

Herein, stomatal plant transpiration inspired the design of a surface structure adjustable wood aerogel island solar evaporator (WAISE) (Figure S1, Supporting Information). WAISE was prepared using a poly (n‐isopropylacrylamide)‐modified multi‐walled carbon nanotube (MWCNT‐g‐PNIPAM) coated wood aerogel/imidazolite zeolite backbone‐67 (WA/ZIF‐67) composite (**Scheme** **1**a). The surface structure of WAISE can be adjusted according to temperature changes to achieve rapid diffusion of surface vapors, while the island structure allows salt gradient rearrangement to make it salt‐resistant (Scheme 1d). The hydrophilicity of the wetted PNIPAM decreased slightly as it warmed up, avoiding the temperature drop caused by excessive water layer coverage of the superhydrophilic aerogel. The introduction of ZIF‐67 as a well hydrophilic metal–organic framework (MOF) material significantly reduced the evaporation enthalpy of water in WAISE and increased the evaporation rate. The adjustable surface benefits from the conversion between the two isomers at different temperatures achieved by poly‐n‐isopropylacrylamide (PNIPAM), which has differences in volume and wettability (Scheme 1b,c). Finally, a high evaporation rate of 3.3 kg m^−2^ h^−1^ (20 wt% NaCl) under one sun was achieved, with no salt accumulation on the surface for 200 h of continuous evaporation. In addition, its evaporators have shown great potential for applications in wastewater purification, thermoelectricity, and irrigation.

## 2 Results and Discussion

### 2.1 Properties and Characterization of Materials

The synthesized PTMs (MWCNT‐g‐PNIPAM) were characterized and analyzed. The surface structure and morphology of the materials were observed by scanning electron microscopy (SEM). The surface of MWCNT‐g‐PNIPAM powder is rougher than that of MWCNT, which favors light scattering (Figure S2a–d, Supporting Information). The aqueous dispersion of MWCNT quickly solidified (Figure S3a, Supporting Information), while the aqueous dispersion of MWCNT‐g‐PNIPAM remained stable suspension without solidification after 48 h (Figure S3b, Supporting Information). The excellent water dispersibility of MWCNT‐g‐PNIPAM may be related to the hydrophilic nature of PNIPAM. MWCNT‐g‐PNIPAM formed more uniform coatings than MWCNT, which was probably due to the modification of PNIPAM, which prevented the self‐aggregation and improved the dispersion ability of MWCNT (Figure S4a,b, Supporting Information). Severe peeling occurred when the ordinary MWCNT‐coated wood aerogel was dropped from a high place or immersed in water, while MWCNT‐g‐PNIPAM did not show any peels (Figure S5a–d, Supporting Information). This indicates that the coating of MWCNT‐g‐PNIPAM on wood aerogel is more robust than the regular MWCNT. A larger diameter of MWCNT‐g‐PNIPAM can be observed from the TEM images compared to MWCNT, which may be due to the MWCNT surface being wrapped by PNIPAM (Figure S6a–d, Supporting Information). The XRD spectrum showed the characteristic peak of PNIPAM at 20.4 for 2θ in MWCNT‐g‐PNIPAM, proving the successful synthesis of MWCNT‐g‐PNIPAM (Figure S7a, Supporting Information**)**.^[^ ^54^ ^]^ The functional groups of MWCNT‐g‐PNIPAM were analyzed by FTIR (Figure S7b, Supporting Information). The characteristic peaks of MWCNT‐g‐PNIPAM at 1460, 1550, and 1645 cm^−1^ were attributed to the C─H bending vibration of the isopropyl group and the N─H stretching vibration of the amide group in PNIPAM, respectively. These results fully demonstrate the successful synthesis of MWCNT‐g‐PNIPAM.^[^ ^55^ ^]^ The elements of MWCNT‐g‐PNIPAM were further characterized by X‐ray photoelectron spectroscopy (XPS). The characteristic peak of N1s appeared in the XPS spectrum of MWCNT‐g‐PNIPAM, which may be from the amide group of PNIPAM (Figure S7c, Supporting Information). The thermal stability of MWCNT‐g‐PNIPAM was characterized by thermogravimetric analysis (TGA). The MWCNT‐g‐PNIPAM exhibits good thermal stability properties, as shown in TGA spectra, and the thermal degradation temperature is high of MWCNT‐g‐PNIPAM (Figure S7d, Supporting Information).

**Figure 1:**
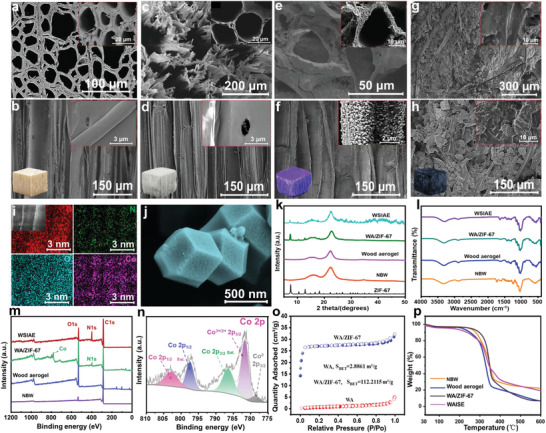
Characterization of NBW, wood aerogel, WAISE. a,c,e,g) Cross‐section picture of SEM. b,d,f,h) SEM image of the longitudinal section. i) EDS mapping images ofthe longitudinal section of WA/ZIF‐67. j) SEM picture of ZIF‐67. k) XRD spectrum. l) FTIR spectrum. m) XPS spectrum. n) High‐resolution XPS spectra of Co 2p. o) N_2_ adsorption‐desorption isotherms. p) TGA spectrum.

The morphology of the evaporator was observed by SEM. Natural balsa wood (NBW) was distributed in micrometer pores in cross‐section (**Figure** **1**a) and small‐sized pores in longitudinal walls (Figure 1b). The connecting channel of NBW serves as a natural water transport path of the evaporator. The pore walls of WA were thinner and more open after removing lignin and hemicellulose (Figure 1c,d). The uniform growth of ZIF‐67 on the cross‐sectional and longitudinal sections of WA/ZIF‐67 indicated good binding of ZIF‐67 to WA (Figure 1e,f and Figure S8a, Supporting Information). The uniform distribution of C, N, O, and Co elements on the surface of WA/ZIF‐67 can be seen from EDS images (Figure 1i). The content of Co elements in WA/ZIF‐67 reached 10.41%, further demonstrating the massive growth of ZIF‐67 in WA (Figure S8b,c, Supporting Information). , The SEM showed that ZIF‐67 was well crystallized and had a dodecahedron shape (Figure 1j and Figure S9, Supporting Information). The distribution of MWCNT‐g‐PNIPAM on the WAISE surface was uniform, and MWCNT‐g‐PNIPAM was tightly bound to WA/ZIF‐67 (Figure 1g,h). The characteristic peak of ZIF‐67 was observed for both WAISE and WA/ZIF‐67 in XRD spectra, proving the successful growth of ZIF‐67 on WA (Figure 1k). The disappearance of the characteristic peaks of cellulose at 1595, 1504, and 1462 cm^−1^ and the characteristic peaks of hemicellulose at 1738 and 1238 cm^−1^ in WA by the FTIR spectrum proved the clean removal of cellulose and hemicellulose in WA (Figure 1l and Figure S10a, Supporting Information). The sharp peak at 425 cm^−1^ of WA/ZIF‐67 is from the Co─N bond on ZIF‐67, which further confirms the successful growth of ZIF‐67 on WA (Figure S10b, Supporting Information).^[^ ^56^ ^]^ The chemical composition and elemental valence of the materials were further characterized by XPS (Figure 1m). The characteristic element Co from ZIF‐67 appears in the XPS spectrum of WA/ZIF‐67. The disappearance of Co elements in WAISE indicated that the MWCNT‐g‐PNIPAM coating tightly wraps WA/ZIF‐67, while the amide group in PNIPAM caused the enhancement of the N element peak. The high‐resolution Co spectra were obtained by fitting Co^0^, Co^3+/2+^, and satellite peaks (Figure 1n). The characteristic peaks of the Co 2p^3/2^ region at 780.4 and 778.6 eV belong to the zero valence state of Co oxide (Co^2+^) and Co, respectively. The characteristic peak at 785.7 eV corresponds to the satellite peak.^[^ ^57^ ^]^ The adsorption and desorption isotherm spectra of nitrogen reveal the extremely high specific surface area of ZIF‐67 (1205.1149 m^2^ g^−1^) (Figure S11, Supporting Information). The specific surface area of WA/ZIF‐67 increased about 39 times compared to WA, resulting from the introduction of ZIF‐67 (Figure 1o). The TGA spectra show that WAISE has good heat resistance (Figure 1p).

### 2.2 Highly Hydrated Polymer Network

**Figure 2:**
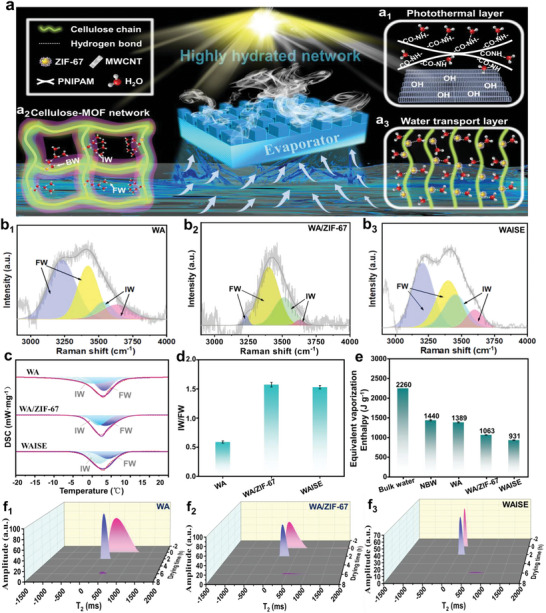
a) WAISE hydration network, (a_1_) is the MWCNT‐g‐PNIPAM, (a_2_, a_3_) are the surface and side of WA/ZIF‐67, respectively. Characterization of the hydration state of WA, WA/ZIF‐67, and WAISE. b_1_–b_3_) Raman spectrum. c) DSC spectrum. d) IW/FW value. e) Equivalent vaporization enthalpy. f_1_–f_3_) Low‐field NMR relaxation spectrum.

Water is usually present in matter as free water (FW), intermediate water (IW), and bound water (BW); the intermediate water requires the least amount of energy.^[^ ^58^ ^]^ The introduction of different materials may lead to changes in functional groups and pore structure, which may result in different hydration degrees.^[^ ^59^ ^]^ Cellulose network, ZIF‐67 all have strong interactions with water, and the amide group of the PNIPAM chainalso easily forms hydrogen bonds with water (Figure 2a_1_‐a_3_). The highly hydrated network of WA/ZIF‐67 and the hydration of the PNIPAM chain led to a redistribution of water states inside the systems. The hydration interactions were investigated by Raman spectroscopy and differential scanning calorimetry (DSC). The peaks in the Raman spectra at 3233 and 3401 cm^−1^ correspond to FW and the peaks at 3514 and 3630 cm^−1^ could be related to IW. FW exhibits four hydrogen bonds, while IW shows weak or non‐hydrogen bonds; thus, IW requires lower evaporation energy than FW.^[^ ^60^ ^]^ There was a significant increase in IW content in WAISE and WA/ZIF‐67, as seen in the Raman spectra. This was probably due to the strong interaction of ZIF‐67 with water resulting in weaker interactions between water molecules, thus increasing the weak state water content (Figure 2b_1_‐b_3_). Thus ithe DSC peaks near 0 °C can be analyzed for the thermal behavior of IW and FW, since bound water (BW) is a strong interaction without heat absorption peaks. The DSC test results were consistent with the Raman spectra, and the IW in WA/ZIF‐67 and WAISE were significantly more than the WA. The values of IW/FW in WA, WA/ZIF‐67, and WAISE obtained by DSC were 0.61, 1.63, and 1.54, respectively (**Figure** **2**c,d). The introduction of the photothermal layer (MWCNT‐g‐PNIPAM) resulted in a slight decrease in the IW content of WAISE, which is due to its lower hydrophilicity than WA/ZIF‐67. The equivalent enthalpy of evaporation of the material was determined using a dark evaporation experiment (Figure 2e; Note S1, Supporting Information**)**. The value of enthalpy was further caculated by equation (1) (Supporting information). The value of enthalpy of bulk water, NBW, WA, WA/ZIF‐67, and WAISE were 2260, 1440, 1389, 1063, and 931 J g^−1^, respectively. The evaporation enthalpy of WAISE was significantly lower compared to that of NBW, which could be attributed to the introduction of ZIF‐67. Evaporators are saturated with water in a short period of time, which indicates that they have good hydrophilicity (Figure S12a‐d, Supporting Information). Wettability of evaporators was further quantified by contact angle testing (Figure S13, Supporting Information). NBW, WA, WA/MOF, and WAISE all have good hydrophilicity and the droplet spreads completely on the surface of the material in a short time. As expected the surface wettability of WAISE was reduced under simulated light conditions due to the conformational change of PNIPAM due to warming. The contact angle of WAISE under operating conditions (wet state and light radiation) was further tested to verify whether the reduction of its hydrophilicity affects water transport. The results showed that the wet state WAISE still has good wettability under light and is slightly worse than the superhydrophilic WA. The suitable wettability of WAISE's surface in operating conditions prevents the covering of the surface with a water layer caused by WA's excessive hydrophilicity, which facilitates surface temperature rise and evaporation.

The hydration state and dehydration capacity of the materials were further evaluated by placing the water‐absorbing saturated materials in an oven (50 °C) to observe the dehydration process. The T_2_ typical relaxation spectrum of the material was recorded by LF‐NMR to analyze the hydration changes during dehydration. A large amount of water was present in WA and WA/ZIF‐67 in the initial state, and the relaxation signal shifted rapidly from FW (T_2_ > 100 ms) to IW (10 ms < T_2_ < 100 ms) and BW (T_2_ < 10 ms) regions within 2 h (Figure 2f_1_,f_2_). In the initial state of WAISE, the signal in the FW region was significantly weaker than WA and WA/ZIF‐67, probably owing to the introduction of MWCNT‐g‐PNIPAM to attenuate the water content of the surface. The relaxation signal of WAISE almost disappeared after 6 h and the WA still had a significant hydration signal (Figure 2f_3_). The above evidence suggests that WAISE dewatering was faster than WA and WA/ZIF‐67. Optical photographs from the drying process show that WA and WA/ZIF‐67 were severely after 6 h in the oven, while WAISE was less deformed than them (Figure S14a, Supporting Information). This may be due to the mechanical stability imparted to WAISE by the MWCNT‐g‐PNIPAM surface layer. The surface was dehydrated before the interior during drying because the surface photo‐thermal layer was hotter. The dried surface layer acts like a fixed structural framework preventing the structural further collapse of the interior structure due to dehydration (Figure S14b, Supporting Information). WA and WA/ZIF‐67 severe deformation lead to channel distortion and aperture blockage, creating a strong adhesion between water and material and severely hindering the water vapor diffusion.^[^ ^42^ ^]^ WAISE maintained a relatively intact channel and open surface pores, greatly improving vapor diffusion and thus speeding up evaporation and dehydration. In addition, WAISE has a low density and high strength. It was placed on the leaf without falling and can withstand more than 50 g of weight (Figures S15 and S16a, Supporting Information). The evaporator can quickly recover its shape after being squeezed after absorbing water, and has good resilience performance (Figure S16b, Supporting Information). The evaporating material also has good plasticity and can be prepared in various shapes (Figure S17, Supporting Information).

### 2.3 Thermal Management and Evaporation Mechanisms

**Figure 3:**
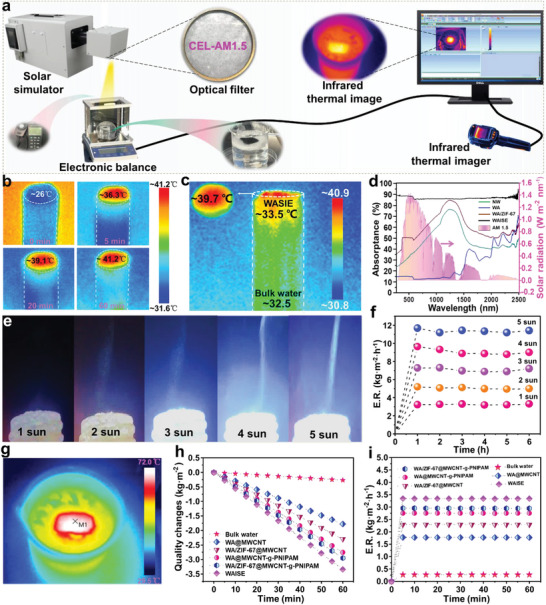
Evaporation performance test of WAISE. a) Schematic diagram of the solar device. b, c) The average surface temperature of the infrared thermography test. d) The UV–Vis‐NIR spectra. e) Optical photographs of evaporation at 1–5 suns. f) Evaporation performance under different solar light intensities. g) Surface temperatures of WAISE in 5 suns. h–i) Change in mass and evaporation rate of salt water for material evaporation in 1 h.

WAISE was fixed with foam and placed in a glass to make an evaporation apparatus for evaporation experiments (**Figure** **3**a; Figure S18, Supporting Information). MWCNT‐g‐PNIPAM has a light absorption of more than 85% and is suitable as PTMs (Figure S19, Supporting Information). MWCNT‐g‐PNIPAM has a structural change due to the increase in temperature under light radiation, making the prepared coating porous at high temperatures. The changes in the MWCNT‐g‐PNIPAM coating structure with temperature were more clearly observed by applying it on the surface of a soft cellulose aerogel. The MWCNT‐g‐PNIPAM coating contracted with the increase in temperature, resulting in a clearly visible porous structure (Figure S20, Supporting Information).

The temperature of WAISE in brine was tested using infrared thermography and its thermal management capability was analyzed. The surface temperature of WAISE with foam protection quickly reached about 43 °C in 20 min, showing the good photothermal properties of the material and the good thermal insulation of the foam (Figure S21, Supporting Information). The thermal transfer analysis was performed by removing the protective foam to further analyze the thermal management capability of WAISE. The surface of the evaporator without foam protection rose from 26.0 to 41.2 °C in 1 h (Figure 3b). The temperatures of the evaporator and bulk water after stabilization (after 3 h) were tested. The surface and side temperatures of WAISE were 39.7 and 33.5 °C respectively, and the bulk water temperature was 32.5 °C (Figure 3c). The surface temperature (39.7 °C) was lower than the maximum temperature (41.2 °C) after temperature stabilization, indicating that the surface was evaporating rapidly to remove some of the heat. The side temperature of WAISE was 6.2 °C lower than the surface temperature, proving its good adiabatic properties. WAISE showed good thermal management with 7.2 °C higher surface temperature than bulk water. The good thermal management is due to the fact that the hydrophilicity of the surface of PNIPAM is lower than that of WA under operating conditions, which contributes to the reduction of heat loss. Meanwhile, the low thermal conductivity of WA also reduces heat loss.^[^ ^61^ ^]^

The results of AFM tests showed that the introduction of ZIF‐67 increased the surface roughness of wood aerogel, which was beneficial in enhancing the light scattering on the surface of the material (Figure S22, Supporting Information). The light absorption of WAISE in the full spectral range (≈90%) was greater than that of NW, WA, and WA/ZIF‐67 (Figure 3d). The enhanced light absorption of WAISE is mainly due to the strong light absorption of MWCNTs‐g‐PNIPAM coating. The rough surface and channels modified by ZIF‐67 further enhanced the light absorption. The evaporation performance of the material was tested under different light intensities. The surface temperature of WAISE reached 70.2 °C under 5 suns (Figure 3g), and the average evaporation rate reached 11.4 kg m^−2^ h^−1^ (Figure 3e,f). Therefore, the evaporation performance increased with the increase of light intensity within a certain range. The evaporation efficiency was obtained by calculating Equation S (2) (Supporting Information) (Notes S2, Supporting Information). The evaporation efficiency of WAISE reached 91.3% under 1 sun (Figure S23, Supporting Information). The evaporation properties of different materials were further investigated. The evaporation rates of bulk water, WA@MWCNT, WA/ZIF‐67@MWCNT, WA@MWCNT‐g‐PNIPAM, WA/ZIF‐67@MWCNT‐g‐PNIPAM (WZMP), and WAISE were 0.27, 1.78, 2.29, 2.75, 2.95, and 3.34 kg m^−2^ h^−1^, respectively, and the addition of MOF and PNIPAM accelerated the evaporation rate (Figure 3h,i). The evaporation rate of WAISE improved by 87.64% over WA@MWCNT.

**Figure 4:**
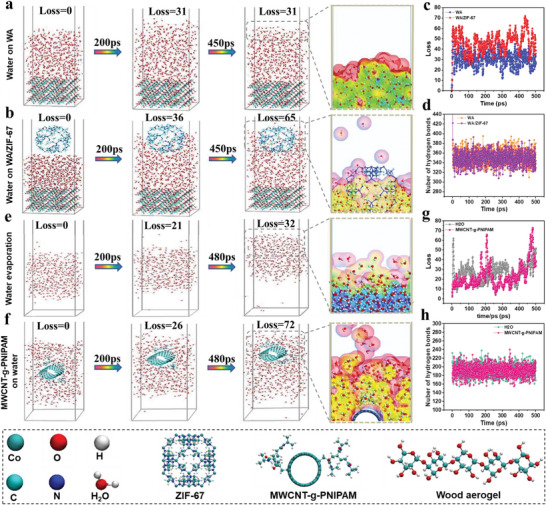
MDS simulations of evaporation processes of WA/water and WA/ZIF‐67/water units, where (a, b) are snapshots of interfacial evaporation at 0, 200, and 450 ps for MDS, and (c, d) are statistics of water molecule loss and number of hydrogen bonds between water molecules within 500 ps. MDS simulations of evaporation processes of pure water and MWCNT‐g‐PNIPAM/water units, where (e, f) are snapshots of interfacial evaporation at 0, 200, and 480 ps, respectively, and (g, h) are statistics of water molecule loss and the number of hydrogen bonds between water molecules within 500 ps.

Reducing the enthalpy of evaporation can effectively reduce the energy demand during the water evaporation process, which is extremely important for increasing the rate of evaporation and freshwater production at a given energy input. The reduction mechanism of the evaporation enthalpy by ZIF‐67 and MWCNT‐g‐PNIPAM was investigated by molecular dynamics simulation (MDS). The hydrogen bonding density and diffusion patterns of water molecules on different surfaces during solar evaporation were also investigated (Note S3, Supporting Information). WA/water and WA/ZIF‐67/water units were established, and the dynamic distribution of water molecules in the two units was simulated (Note S3a and Movie‐S1, Supporting Information). The number of escaped water molecules and the number of hydrogen bonds between water molecules during the evaporation process were quantified. At 0 ps, 500 water molecules were stationary in the presence of WA and WA/ZIF‐67 surfaces . The number of water molecules lost from the WA/ZIF‐67 surface was 36 (i.e., evaporation), slightly more than WA (31) within 200 ps. The WA/ZIF‐67 surface water molecules had 65 molecules escaping into the gas phase, significantly higher than the WA (31) within 450 ps (Figure 4a, b). The loss of water molecules in the unit with the addition of ZIF‐67 in 500 ps was greater than in the system with WA alone (**Figure** **4**c). Water molecules gradually wrapped around the ZIF‐67 surface and entered the pore channel during evaporation, confirming the existence of an extremely strong interaction between hydrophilic ZIF‐67 and water. The strong interaction between ZIF‐67 and water molecules greatly interfered with the hydrogen bonds between water molecules, resulting in a decrease in the density of water‐water hydrogen bonds (Figure 4d). Therefore, WA/ZIF‐67 can provide a higher evaporation rate with the same energy input. Similarly, the effect of MWCNT‐g‐PNIPAM coating on the water evaporation process was analyzed by MDS (Note S3b and Movie S2, Supporting Information). The evaporation process Water and MWCNT‐g‐PNIPAM/water units were simulated to exclude the interference of WA and ZIF‐67. The loss of water molecules from the surface of the MWCNT‐g‐PNIPAM/water unit within 200 ps was 26 (i.e., evaporation), slightly greater than the evaporation of water 21 . There were 72 water molecules escaping from the surface of the MWCNT‐g‐PNIPAM/water unit into the gas phase within 480 ps, which was more than twice the 32‐escaping amount of water unit (Figure 4e, f). The number of water molecules escaping from the MWCNT‐g‐PNIPAM/water unit within 500 ps was higher than the water unit (Figure 4g). The above results suggested that the introduction of MWCNT‐g‐PNIPAM increased the evaporation rate. Water molecules gradually wrapped around PNIPAM chains during the simulated evaporation of Figure 4f, illustrating that PNIPAM had a good binding ability with water molecules. This also demonstrated that the PNIPAM was still mostly hydrated above the lowest critical solubilization temperature (LCST).^[^ ^62^ ^]^ Hydrogen bonding forces between PNIPAM chains and water molecules weakened hydrogen bonding forces between water molecules. The binding of PNIAPM chains to water molecules allowed the production of more intermediate water around them, thus reducing the evaporation enthalpy of water. The hydrogen bond density calculations showed that the water–water hydrogen bond density of the MWCNT‐g‐PNIPAM/water unit was lower than that of the water unit, which was consistent with the above conclusions (Figure 4h). Therefore, the addition of MWCNT‐g‐PNIPAM can provide higher evaporation rates at the same energy input. In conclusion, both ZIF‐67 and MWCNT‐g‐PNIPAM can contribute to increase the evaporation rate of the evaporator.

### 2.4 Analysis of Salt Resistance Mechanism

**Figure 5:**
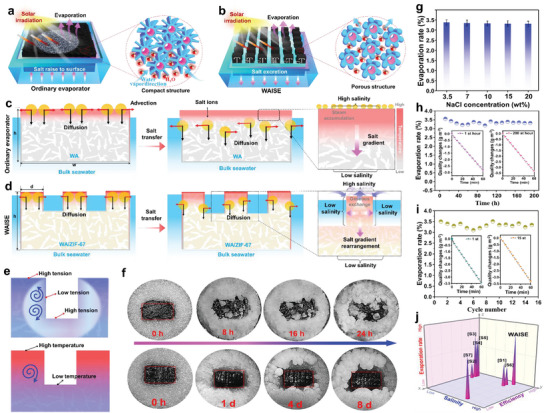
Salt resistance performance analysis of OE and WAISE. a) Structure schematic of OE. b) Structure schematic of WAISE. c) The salt transport paths of OE. d) The salt transport paths of the WAISE. e) Water velocity enhancement mechanism in the fluted structure. f) Snapshot of the evaporation process of OE (top) and WAISE (bottom). g) Evaporation rate of WAISE at different salt concentrations. h) The 200‐hour continuous evaporation test. i) The 15 cycles evaporation test. j) Comparison with similar working performance.

The structure of an ordinary evaporator (WA/ZIF‐67@ MWCNT) is shown in **Figure** **5**a. Ordinary evaporator (OE) has a dense surface structure due to the self‐aggregation of MWCNT. The WAISE has a surface structure with adjustable thermal response compared to OEs (Figure 5b). Salt resistance performance test results showed that WAISE had better salt resistance than OE. The surface of the OE was almost covered with salt crystals within 24 h (20 wt% NaCl) (Figure 5f). The dense surface structure of the OE prevented enough water to the surface, resulting in salt crystallized at the evaporation interface not returning to the bulk water. The WAISE had no salt accumulation and no significant decrease in evaporation rate in different concentrations of saline water (Figure 5g). WAISE has created a record of at least 200 hours of continuous operation without salt accumulation in 20% salt solution (Figure 5h). WAISE also has good cycling stability, and the evaporation performance did not degrade significantly in 15 cycles (Figure 5i).

The diffusion process of salt ions in a normal evaporator and WAISE was simulated, and the mechanism of salt resistance of WAISE was further analyzed. The evaporation at the interface of the evaporator led to higher ion concentrations and densities on the surface, which created gradients of ion concentration and density between the upper and lower portions of the evaporator. The salt transport was achieved due to the creation of the above‐mentioned gradient, which led to the downward diffusion (black arrows), and advection (red arrows) of the brine. The accumulation of salt crystals inside the evaporator can only be avoided when the salt ions in a normal evaporator diffuse to the outside of the materials driven by the chemical potential (Figure 5c). For the WAISE, the increased temperature leads to the contraction of the PNIPAM chain of evaporation surface to produce porous structures (Figure 5d). At the same time, the island‐shaped structure recesses were filled with bulk seawater, which led to the rearrangement of salt concentration gradients (pink arrows). The diffusion of high salt concentration distribution areas inside the island structure to the low salt bulk seawater in the surrounding trough greatly reduced the distance of salt ion diffusion into the bulk seawater (i.e., d<w). The open surface pore structure of WAISE allowed bulk water to reach the surface and flush salt crystals easily.

In addition, the good salt tolerance of WAISE was associated with the production of the Marangoni effect. Marangoni effects include both the solutal and thermal Marangoni effect. The solutal marangoni effect is caused by the generation of surface tension gradients (Figure 5e). The solute (salt) concentration on the upper surface of the island type is higher than on the sides of the island structure, which leads to the creation of a surface tension gradient in the flutes. Thus, the solute marangoni effect occurs in the recesses accelerating the water convection. The temperature Marangoni effect is due to the temperature difference. The light radiation leads to an increase in temperature on the upper material surface, and the heat energy is transferred down. However, the temperature difference between the surface and the interior of WA is large due to its low thermal conductivity. The large temperature difference leads to the temperature Marangoni effect, which accelerates the convection of water in the recess and enhances the rate of surface salt reflux. In addition, the porous structure of the WAISE surface accelerated vapor convection near the surface (blue arrows), preventing vapor accumulation near the surface and accelerating evaporation. In summary, the island structure and open porous surface of WAISE synergized to provide strong salt resistance and high evaporation performance. Notably, WAISE has a record salt tolerance compared to previous biomass evaporators (Figure 5j). Hence, WAISE overcomes the paradox that rapid evaporation and salt accumulation are difficult to balance.

### 2.5 Water Purification and Design of Power Generation Irrigation System

**Figure 6:**
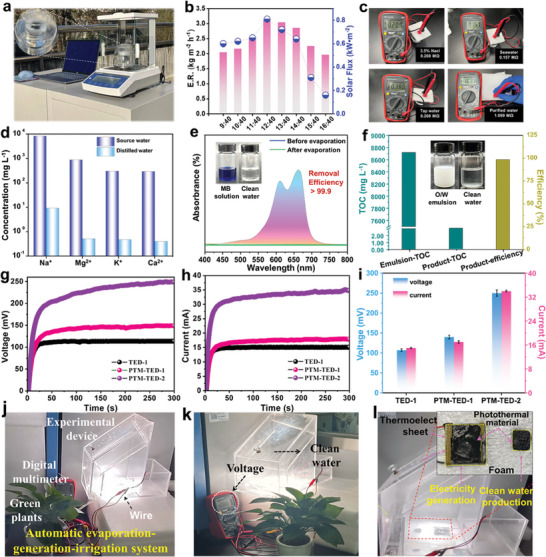
WAISE for desalination, power generation, and irrigation. a) Outdoor freshwater collection experiments. b) Desalination performance in one day outdoors. c–d) are the resistance value and ion concentration test of water e) UV spectrum of methylene blue. f) Performance testing of purification emulsions. Power generation performance tests of TED‐1, PEM‐TED‐1, and PEM‐TED‐2, g) open‐circuit voltage, h) current curve, and i) maximum voltage‐current value. l) Photo diagram of evaporation‐generation‐irrigation system (EGIS) working process.

The desalination and water purification capability of WAISE under natural conditions were evaluated by outdoor experiments using a homemade evaporation device (**Figure** **6**a). The evaporation performance was tested in an outdoor environment from 9:40 a.m. to 16:40 p.m. The maximum evaporation rate reached 3.14 kg m^−2^ h^−1,^ slightly lower than the indoor experiment, which may be caused by the difference in the outdoor ambient temperature and humidity (Figure 6b). The water quality was evaluated by measuring the ohmic resistance and major ion concentration. The ohmic resistance of water was measured by using a multimeter with fixed electrode spacing (Figure 6c). The significantly increased electrical resistance of purified water solutions proves that they have a lower concentration of ions than salt water, seawater, and tap water. The inductively coupled plasma‐optical emission spectrometer results show that the concentration of K^+^, Ca^2+^, Na^+^, and Mg^2+^ ions in natural seawater (from the East China Sea) after purification. This result proves that the purified water meets the drinking water standards of the World Health Organization (WHO) (Figure 6d). This is further evidence that the purified water has good water quality. The water purification capacity of the evaporator was fully evaluated using dye wastewater and surfactant‐stabilized oil‐containing emulsion wastewater (Figure 6e,f). The removal efficiency of the evaporator exceeded 99.9% for both methylene blue dye and emulsion wastewater, which indicated that the evaporator could effectively remove dyes and organic matter from water. The above results prove that WAISE has a strong water desalination and purification capability for wastewater. The excellent photothermal properties of MWCNT‐g‐PNIPAM can generate a large temperature with water. Temperature differences on both sides of the thermoelectric device (TED) can generate electrical energy due to the Seebeck effect, which inspired the design of photothermal materials‐thermoelectric devices (PTM‐TED) by useing temperature differences to generate electricity.^[^ ^63^, ^64^ ^]^ A PTM–TED device was designed by using MWCNT‐g‐PNIPAM as a PTM in combination with a TED (Figure S24a‐d, Supporting Information). The thermoelectric performance of the device was studied under 1 sun (PTM‐TED‐1) and 2 suns (PTM‐TED‐2), respectively. The thermoelectric performance of a TED under 1 sun (TED‐1) was also tested as a control. The photothermal effect of PTM‐TED was shown to be better than the TED by infrared thermograms and temperature data (Figure S24e,f, Supporting Information). The open‐circuit voltage and short‐circuit current test results showed that the current and voltage values of PTM‐TED were significantly higher than those of TED under the same light intensity (Figure 6g‐i). The voltage and current values of PTM‐TED increased with the enhancement of light radiation. A light‐driven automatic evaporation‐generation‐irrigation system (EGIS) was designed in situ based on the water purification capability of WAISE and the power generation capability of PTM–TED (Figure S25, Supporting Information). The seawater was poured into the reservoir and the water automatically flowed into the working tank as the water level rose. The seawater flowed into the working tank to float the PTM‐TED to the water's surface. The system started to work under the simulated sunlight drive and the universal meter voltage value rose rapidly within a short period. The vapor from the top and side walls of the unit condensed over time into clean water to irrigate the greens through the water delivery tanks and pipes. The EGIS exhibits great potential for practical applications through up to 3 days of continuous irrigation and voltage output (Figure 6j–l; Figure S26, Supporting Information).

## 3 Conclusion

MWCNT‐g‐PNIPAN, with the reversible transformation of contraction–expansion, was synthesized and developed as a solar evaporator with adjustable and thermally responsive surface structure by a coating method and surface structure design. The unique thermally induced adjustable surface gives this evaporator excellent characteristics such as a high evaporation rate, good salt tolerance, good thermal management, and sufficient moisture supply. The adjustable surface and island structure synergistically increase the evaporation rate of WAISE by 87.64% over conventional WA evaporators, and a record 200 h continuous evaporation surface without salt crystallization in highly concentrated brine (20% NaCl). In addition, WAISE exhibits superb water purification capability for efficient purification of seawater, organic emulsion wastewater, and dye wastewater. An evaporation‐generation‐irrigation unit was developed to operate continuously for at least 3 days. Therefore, based on the synergy of smart materials and surface structure engineering, this developed evaporator provides a new perspective to improve solar evaporation performance further. It provides a theoretical basis for studying the hydration state and evaporation mechanism of wood/MOF‐based evaporators. It also creates a new idea for the multifunctional and intelligent design of future solar evaporation systems.

## Conflict of Interest

The authors declare no conflict of interest.

## Supporting information

Supporting Information

Supplemental Movie 1

Supplemental Movie 2

## Data Availability

The data that support the findings of this study are available in the supplementary material of this article.
