# Supplementary material for: Multifunctional Solar Evaporator with Adjustable Island Structure Improves Performance and Salt Discharge Capacity of Desalination
Source: Adv Sci (Weinh). 2023 Oct 24;10(35):2305523. doi: 10.1002/advs.202305523 (PMC10724399; doi:10.1002/advs.202305523)
Supplement: Supplementary file 1 — Supporting Information [file ADVS-10-2305523-s003.pdf]

ADVANCED SCIENCE
Open Access

Supporting Information

for *Adv. Sci.*, DOI 10.1002/advs.202305523

Multifunctional Solar Evaporator with Adjustable Island Structure Improves Performance and Salt Discharge Capacity of Desalination

Jianfei Wu, Ziwei Cui, Yang Yu, Bo Yue, Jundie Hu, Jiafu Qu, Jianzhang Li, Dan Tian* and Yahui Cai**

Supplementary Information for

Multifunctional solar evaporator with adjustable island structure improves performance and salt discharge capacity of desalination

Jianfei Wu,[1] Ziwei Cui,[1] Yang Yu,[1] Bo Yue,[1,2] Jundie Hu,[3] Jiafu Qu,[3] Jianzhang Li*[1,4]

Dan Tian,[1]* Yahui Cai,*[1,5]

[1] Co-Innovation Center of Efficient Processing and Utilization of Forest Resources, College of Materials Science and Engineering, Nanjing Forestry University, Nanjing 210037, PR China

[2] School of Chemical and Pharmaceutical Engineering, Qilu University of Technology (Shandong Academy of Sciences), Jinan 250353, PR China.

[3] School of Materials Science and Engineering, Suzhou University of Science and Technology, Suzhou 215009, PR China

[4] Key Laboratory of Wood Material Science and Application (Beijing Forestry University), Ministry of Education, Beijing 100083, China

[5] Dehua Tubaobao New Decoration Material Co., Ltd., Huzhou 313200, PR China

Supplementary Information Contents

S1. Materials and Methods

S2. Supplementary Notes

S3. Supplementary Figures

S4. Supplementary Tables

S5. Supplementary Movie

S6. Supplementary References

S1. Materials and Methods

Material

Balsa wood with a density of about 0.112 g·cm^{-3} purchased from Zhuhai, Guangdong was used in this work, and the samples were cut into cubes with dimensions of 10×10×10 (mm). All drugs were purchased from Sinopharm Chemical Reagent Co., Ltd, China, and were not further processed. The drugs used include Hydrochloric acid (HCl, 37%), Nitric acid (HNO_3, 67%), Sulphuric acid (H_2SO_4), Multi-walled CNT, Trichlorovinylsilane (TVS, $CH_2CHSiCl_3$), Toluene (≥99.5), Sodium chlorite ($NaClO_2$, 80%), Acetic acid (CH_3COOH, 36%), Sodium hydroxide (NaOH, ≥96), Cobalt nitrate hexahydrate ($Co(NO_3)\cdot 6H_2O$, 99%)， 2-Methylimidazole (2-MeIm, $C_6H_6N_2$, 97%), methanol anhydrous (CH_3OH, ≥99.5%), sodium chloride (NaCl, ≥99.5%), Polyvinyl pyrrolidone (PVP), $(C_6H_9NO)_n$, Ethanol (C_2H_5OH, ≥99.7%), 2,2'-azodi(isobutyronitrile) (AIBN), ($C_8H_{12}N_4$, 98.0%), N-isopropylacrylamide (NIPAM), N,N-dimethylformamide (DMF).

Methods

Preparation of MWCNT-g-PNIPAM

MWCNT was pretreated with aqueous HCl (0.1 M) and sonicated for 3 h at room temperature to remove surface impurities and produce hydroxyl groups on the MWCNT surface. The dried product was immersed in a beaker containing a solution of trichlorovinylsilane (TVS, 1 g) and toluene (49 g) for 12 h at room temperature. The samples were removed from the solution, washed three times with stirring in toluene (25 mL), and then dried in a vacuum oven for 2 h to obtain TVS modified

MWCNT (TVS-MWCNT). TVS-MWCNT was immersed in a mixture consisting of NIPAM (0.375 g), AIBN (0.0082 g) and DMF (45 mL), and the mixture solution was deoxygenated with argon gas and heated in an oil bath at 70°C for 12h. Finally, the powder was washed by centrifugation with a large amount of toluene and subsequently dried under vacuum at 70 °C for 30 min to obtain MWCNT-g-PNIPAM powder. The mechanism of MWCNT-g-PNIPAM synthesis is shown in **Supplementary Figure 27**.

Preparation of WA

NBW was placed in an aqueous solution of $NaClO_2$ (2 wt%) at 80°C for 24 h to delignify lignin (pH adjusted to 4.7 by acetic acid). The materials were washed in deionized water (80 °C) and dried in a desiccator (80 °C). The dried material was placed in NaOH (8 wt%) aqueous solution for 9 h to remove hemicellulose, and then the material was placed in deionized water and washed thoroughly (**Supplementary Figure 28**). The cleaned material was frozen in a -40 °C refrigerator for 10 h and then transferred to a freeze dryer for 48 h (at -60 °C) to obtain wood aerogels.

Preparation of ZIF-67 and WAISE

ZIF-67 was prepared by pouring $Co(NO_3)_2 \cdot 6H_2O$ (4.36 g, 15 mmol) and 2-methylimidazole (4.92 g, 60 mmol) into a solution containing 150 mL of methanol and stirring for 6 h. The ZIF-67 powder was obtained by washing with methanol five times and drying. The mechanism of ZIF-67 synthesis is shown in **Supplementary Figure 29a**.

Wood aerogel/ZIF-67 was prepared by improving the methods already reported.

Specifically, 15 mmol of $Co(NO_3)_2 \cdot 6H_2O$ and 60 mmol of 2-Methylimidazole were poured into solution A and solution B containing 150 mL of methanol, respectively. WA was immersed in solution A and 167 mg of polyvinylpyrrolidone (PVP) was added to enhance the binding of Co^{2+} to WA. In order to promote more Co^{2+} into the interior of WA, the impregnation was done by vacuum pumping for 30 min, and then put into gas to stand for 30 min at atmospheric pressure, and the process was cycled 2 times. The solution B was slowly poured into A after standing under ambient pressure for 5 h. The above method was used to coordinate the 2-MI organic ligand with Co^{2+}. The mixture was stirred on a thermostatic shaker at 120 rpm for 10 h and then the WA samples were removed and washed with ethanol/water (10/90,v/v) solution. Finally, the ZIF-67@WA composites were obtained by freeze-drying for 24 h.

0.2 g of MWCNT-g-PNIPAM powder was evenly dispersed in 2 mL of deionized water solution and sonicated for 10 min to obtain MWCNT-g-PNIPAM aqueous dispersion. The dispersion was evenly coated on the surface of WA/ZIF-67 to obtain WA/ZIF-67@MWCNT-g-PNIPAM intelligent wood-based evaporator (**Supplementary Figure 29b**). The evaporator samples were frozen in a refrigerator at -40℃ for 4h, and then dried in a freeze dryer for 6h. The robust evaporator was moistened to become flexible and WAISE was obtained by surface grooving (**Supplementary Figure 30 and Supplementary Figure 31**).

Fabrication of EGIS

An aqueous dispersion of PTMs (MWCNT-g-PNIPAM) at 0.1 g/ML were dispensed and coated on the surface of a commercial thermoelectric device (TED), and the

photothermal material-thermoelectric device (PTM-TED) was obtained by baking under a simulated light source. The PTM-TED was fixed to the foam and a absorbent paper was attached to the water underneath it (the absorbent paper is used to absorb water as a cold side). The WAISE was fixed on the other side of the foam on the basis of the above to obtain evaporation-generation units. The water collection device was designed and prepared, and the evaporation-generation units was placed in it to obtain EGIS.

Preparation of cellulose aerogel

Cellulose aerogels were prepared with reference to the literature [S1].

Solar steam generation measurement

The prepared evaporation material (20×12×10 mm^3) was fixed in the foam for evaporation experiments. The experiments were performed under constant temperature and humidity laboratory conditions (24 °C, RH=60%). The sunlight was simulated using a xenon lamp light source (Beijing CEL-HXF300-T3, AM 1.5G). The solar flux was measured with a light power meter (CEL-NP2000-2A). The change in mass of water was measured using a balance with an accuracy of 0.1 mg.

Characterize

The scanning electron microscopy (SEM) images and Energy-dispersive X-ray spectroscopy (EDS) mapping images of the wood aerogels before and after modification were observed using field-emission scanning electron microscopy (Hitachi, Regulus 8100, Japan). Transmission Electron Microscope (TEM) images

were measured by a High-resolution TEM (JEM-2100 UHR, Japan). Atomic Force Microscopy (AFM) images were captured by AFM (Dimesion Edge, Germany). The chemical composition of materials investigates by X-ray photoelectron spectroscopy (ESCALAB 250Xi, America). The Contact Angle (CA) Tester (DSA30, US) was used to analyze material wettability. The functional groups were texted by infrared spectrometer (FTIR) (VERTEX 80V, Germany). The main ion concentration in water measured by Inductively Coupled Plasma-Optical Emission Spectrometer (ICP-OES). Voltage and current data were tested with an electrochemical workstation (CHI760E, China) and a multimeter.

S2. Supplementary notes

Supplementary notes 1: dark evaporation experiments

The change in evaporation enthalpy is only related to the content of free and intermediate water due to the high hydration of the wood-based aerogel evaporator. The true evaporation enthalpy of water in the evaporator can be estimated using dark evaporation experiments. The tested evaporators had the same surface area and they were sealed in a dark container of supersaturated potassium carbonate solution. A sample of pure water with the same area is also put into the device for evaporation at the same time for each test. The equivalent enthalpy of evaporation of water in the wood evaporator (E_{equ}) can be calculated by the following equation:

$$E_{equ}m_1 = E_w m_w \quad \textbf{(Supporting Equation 1)}$$

where E_{equ} represents the equivalent evaporation enthalpy of water in the evaporator, m_1 is the mass change of water in the evaporator, E_w is the evaporation enthalpy of pure water, and m_w represents the mass change of pure water during evaporation.

Supplementary notes 2: Calculations of evaporation efficiency

The solar steam evaporation efficiency (η) is defined as:

$$\eta = \frac{\dot{m}\,(H_{pc} + H_s)}{C_{opt}} = \frac{\dot{m}\,(H_{pc} + C\Delta T)}{C_{opt}} \quad \textbf{(Supporting Equation 2)}$$

Where η is the steam evaporation efficiency, $\dot{m}$ denotes the net water evaporation rate under the corresponding solar illumination intensity (deduct the rate of evaporation under dark conditions), H_{pc} is the latent heat of water (i.e., the equivalent enthalpy of evaporation in the evaporator), H_s represents the sensible heat of water evaporation, C_{opt} is the simulated solar intensity on the top surface of the evaporator,

C is the specific heat capacity of water (4.2 kJ^{-1} K^{-1}), and ΔT is the value of the temperature difference at the absorbing surface in one hour.

Supplementary Notes 3: Simulation calculation steps of MDS

In this paper, all molecular dynamics simulations were performed using Gromacs software for kinetic calculations, visualized using VMD, and UFF force fields were selected for kinetic calculations to explain the intermolecular interactions. In addition, the water molecule density number were determined using the Forcite module in the Materials Studio 2018 program of BIOAccelrys.

(a) System of WA/water and WA/ZIF-67/water:

The modeling part uses Packmol software, and a 27 Å × 27 Å periodic simulation pool was established to simulate the evaporation process. Firstly, the wood molecules (A cellulose chain consisting of five monomers was used) are spread and fixed on the bottom surface of the simulated pool, and then a free water layer (500 water molecules, with a density of 1 g/cm^3) is added to the wood surface. To prevent molecular overlap, a vacuum layer of 2 Å is added between water and wood. This model is used as the initial model for wood/water, with ZIF67 added above the free water layer as the initial model for wood/ZIF/water. The model image is shown in the support information.

After minimizing the energy of the model, molecular dynamics simulations were run. Using the temperature control method of Nose, the temperature is controlled at 316 K, and the cutoff radius is selected as 12.5 Å. Under the NVT ensemble, perform a dynamics simulation with a step length of 1 fs, and totaling time is 500 ps, to ensure

that the dynamic results are balanced. Use code to calculate the relationship between the amount of hydrogen bonds and water molecules evaporated in the system and time.

(b) System of water and MWCNT-g-PNIPAM/water:

The modeling part adopts packmol, considering the periodicity of carbon nanotubes, and establishes a cross-section of 25 Å × 23.7 Å simulation pool is used to simulate the evaporation process. Fix the CNT with PNIPAM branches (polymerization degree is 3), and then pack the free water layer (500 water molecules with a density of 1g/cm^3) near the CNT. This model serves as the initial model for CNT/water. Additionally, establish a cross-section of 25 Å × 23.7 Å simulation pool of 23.7 Å is placed with a free water layer as a control. All simulation pools have 200 Å heights to simulate the evaporation environment.

S3. Supplementary Figures

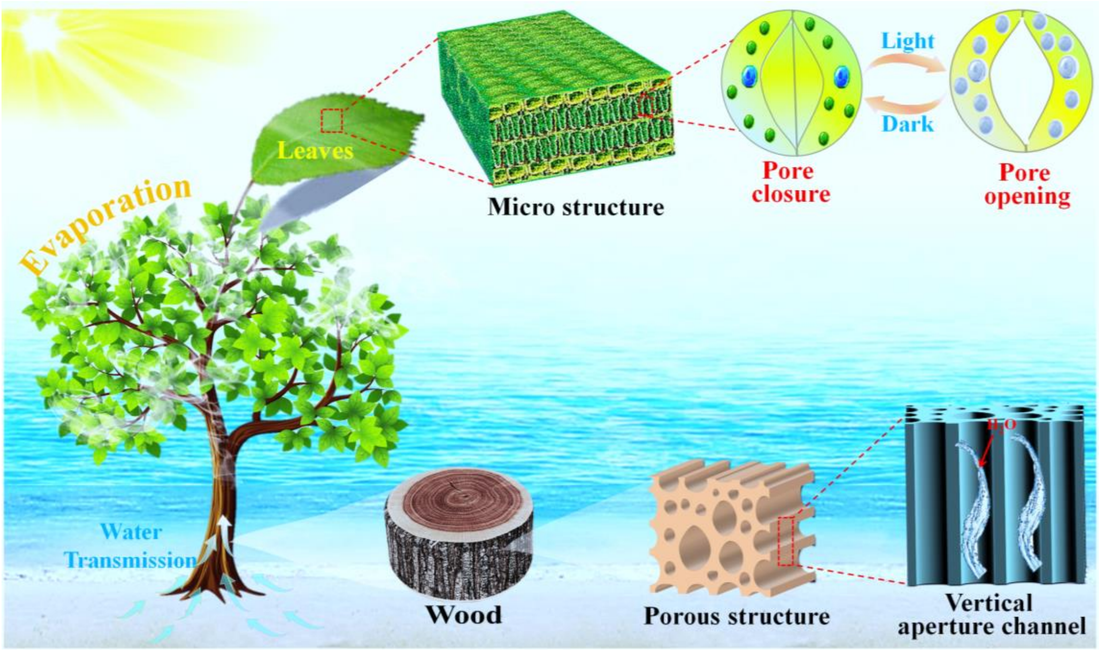

Supplementary Figure 1. Tree channels transport water upward by capillary forces. The stomata of the leaves, on the other hand, open in the light for transpiration, and close in the dark as a reversible process.

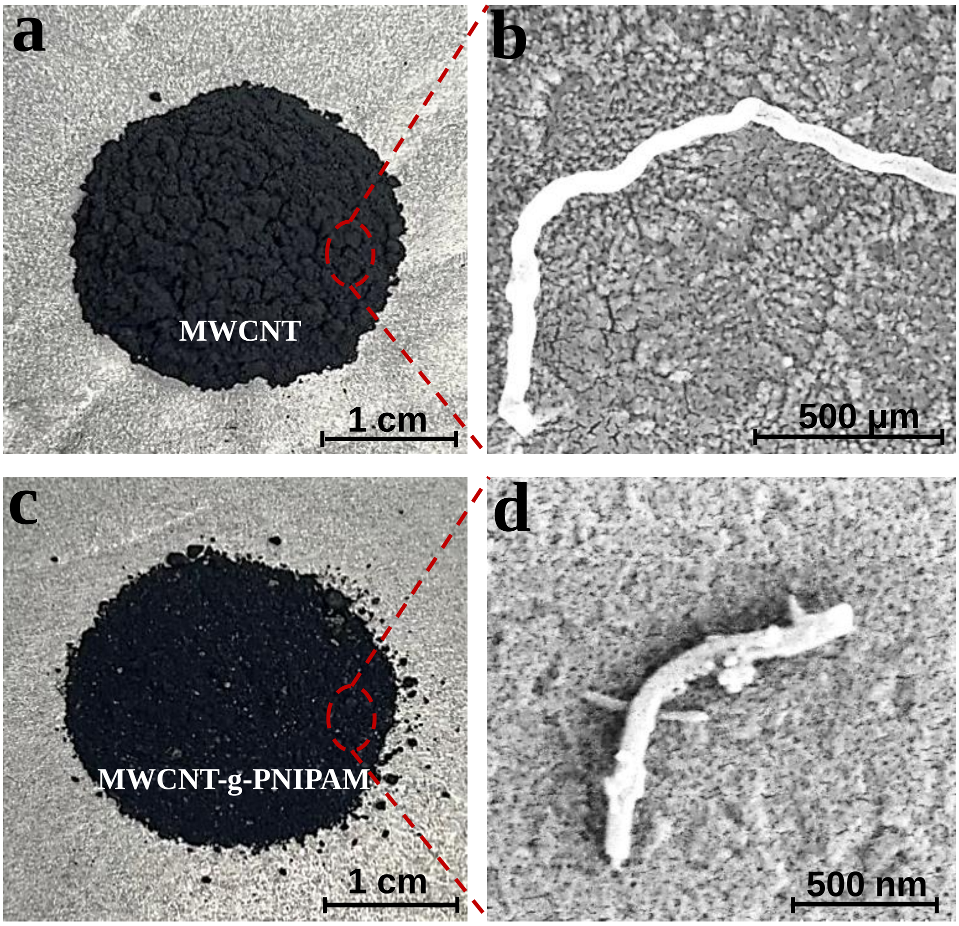

Supplementary Figure 2. MWCNT and MWCNT-g-PNIPAM powders and the corresponding SEM images. (a) MWCNT powders. (b) The SEM image of MWCNT. (c) MWCNT-g-PINPAM Powders. (d) The SEM image of MWCNT-g-PINPAM.

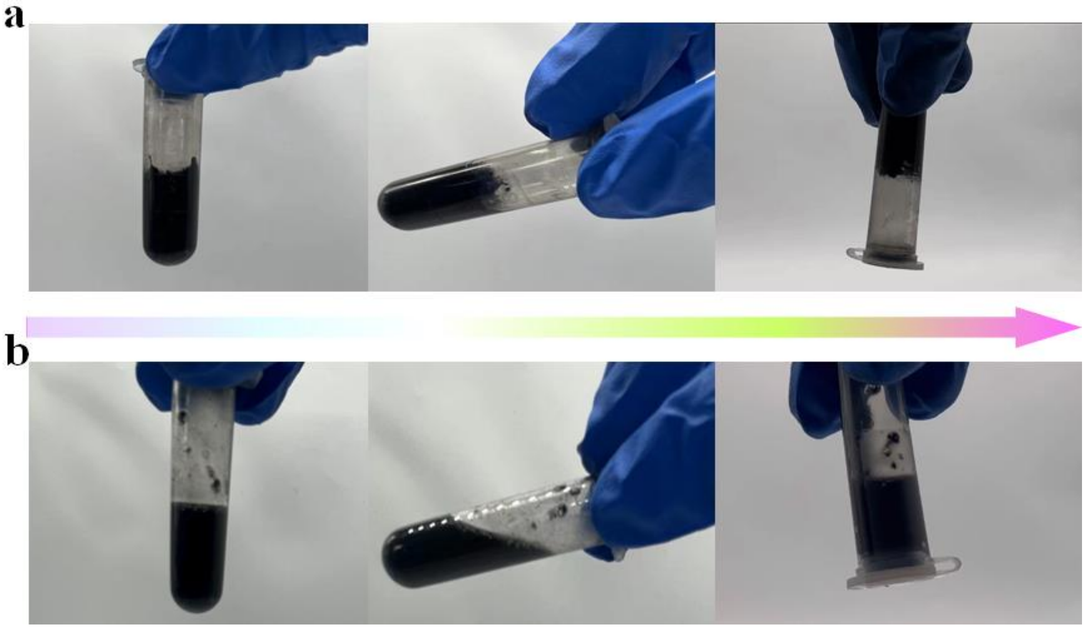

Supplementary Figure 3. Aqueous solutions of MWCNT and MWCNT-g-PNIPAM. (a) The MWCNT aqueous solution self-aggregated and solidifies in a short time. (b) MWCNT-g-PNIPAM maintained a uniform suspension in a flowable state.

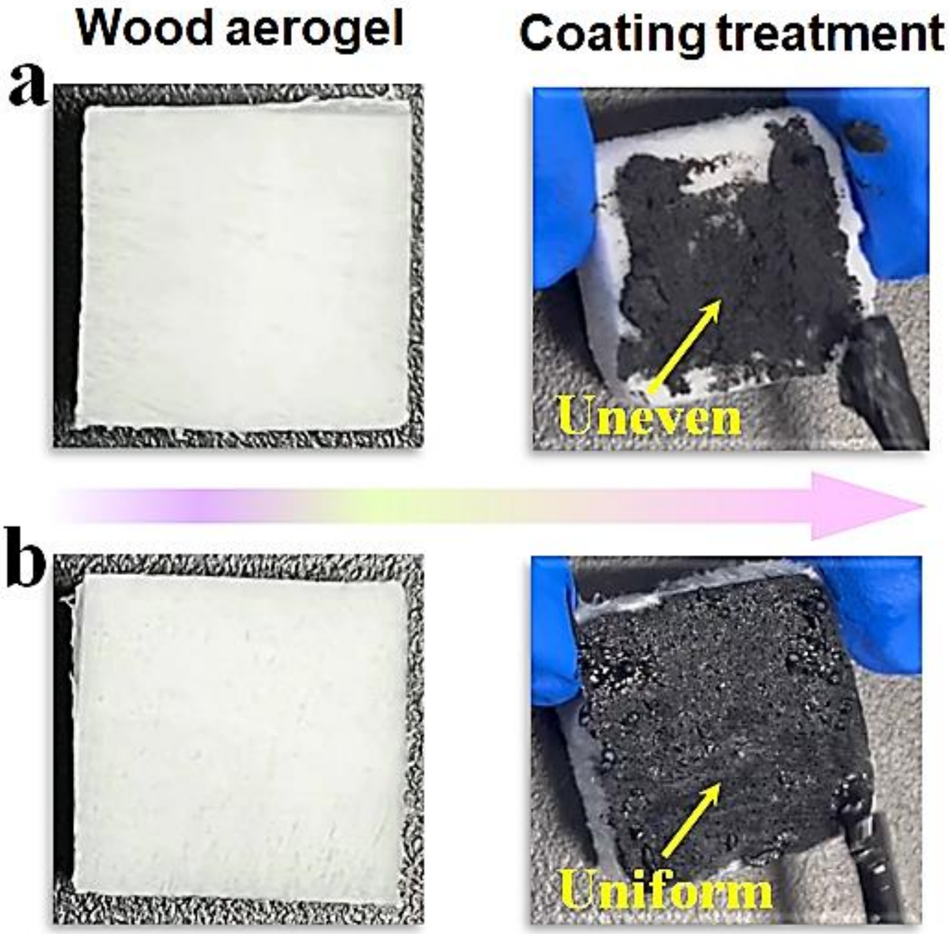

Supplementary Figure 4. Different coating effected on WA surface. (a) MWCNT coating. (b) MWCNT-g-PNIPAM coating.

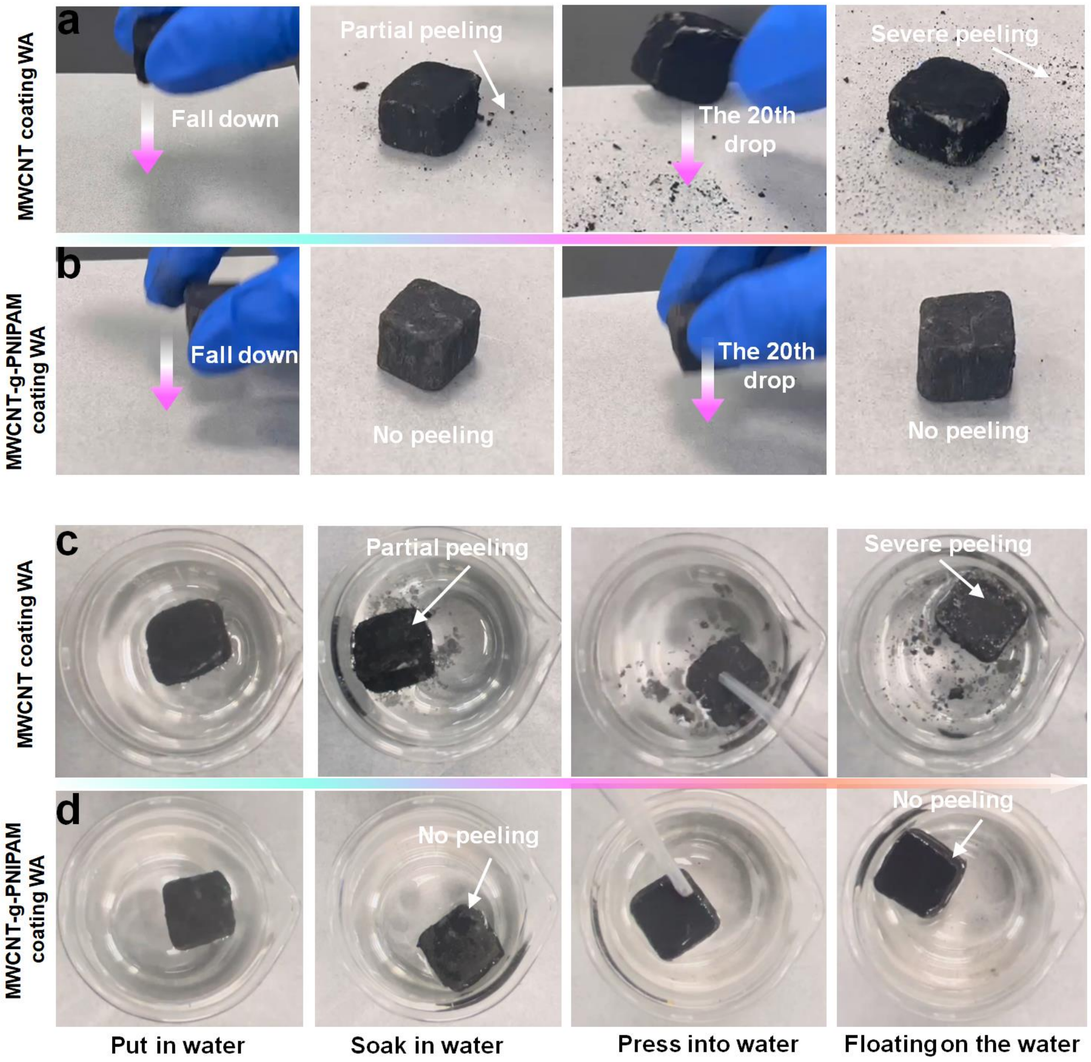

Supplementary Figure 5. Stability tests of the coatings. (a) and (b) are 20 free-falls of MWCNT and MWCNT-g-PNIPAM at a certain height and observing whether the coatings are peeled off or not. (c) and (d) are the submergence of MWCNT and MWCNT-g-PNIPAM into water, respectively, and observing whether the coatings are peeled off or not.

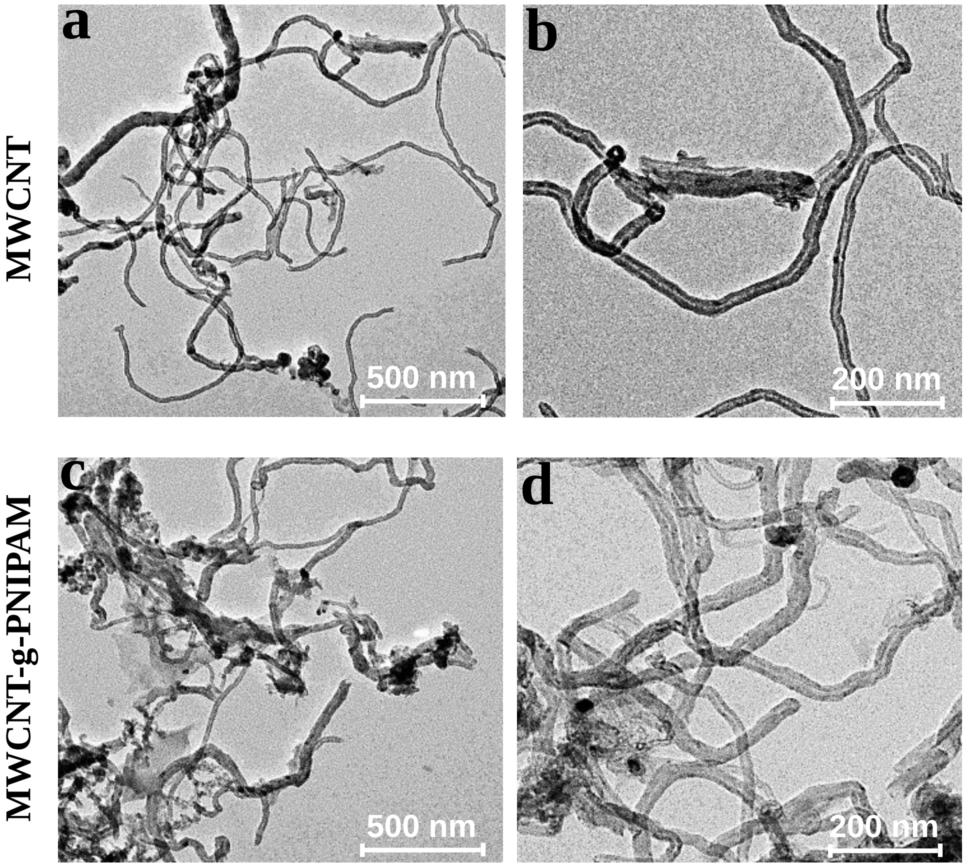

Supplementary Figure 6. TEM images of MWCNT and MWCNT-g-PNIPAM. (a, b) The TEM images of MWCNT. (c, d) The TEM images of MWCNT-g-PNIPAM.

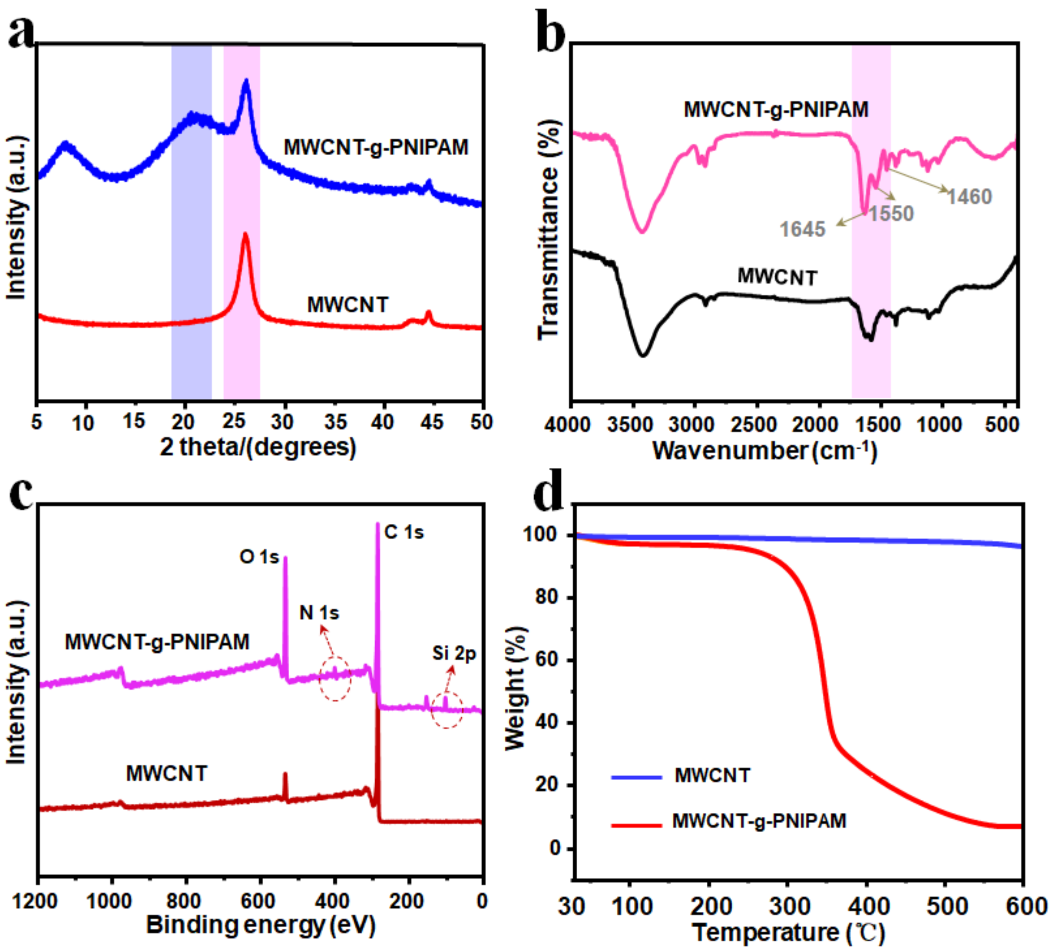

Supplementary Figure 7. Performance characterization of MWCNT and MWCNT-g-PNIPAM. (a) XRD spectra. (b) FTIR spectra. (c) XPS spectra. (d) TGA spectra.

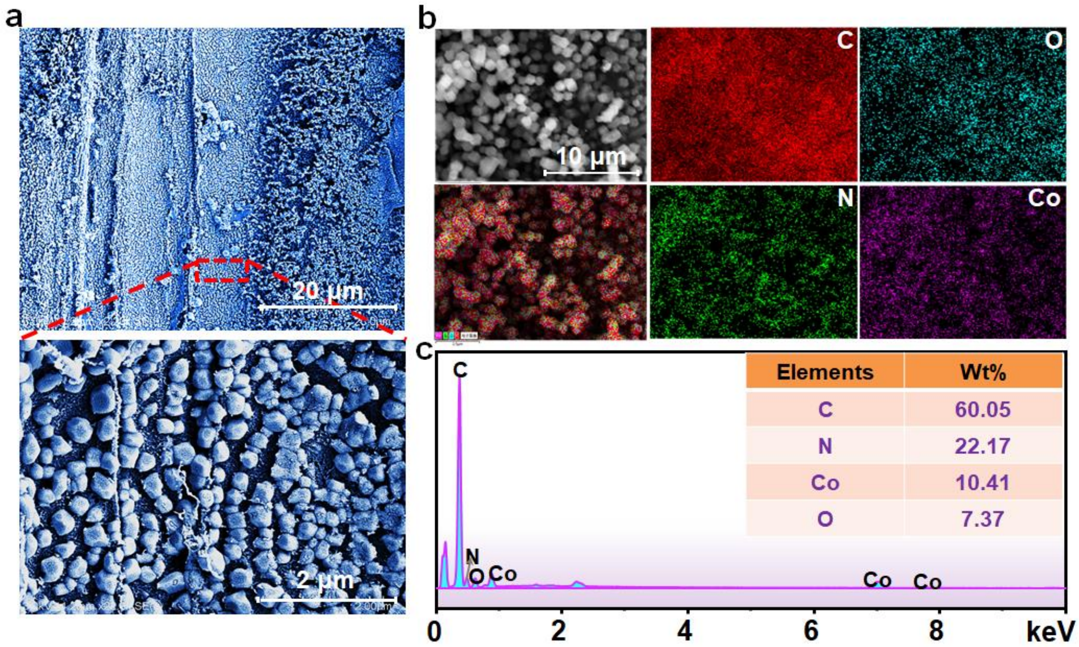

Supplementary Figure 8. (a) The SEM image of the longitudinal section of WA/ZIF-67. (b) EDS mapping images of WA/ZIF-67 surface. (c) The elemental content distribution on the surface of WA/ZIF-67.

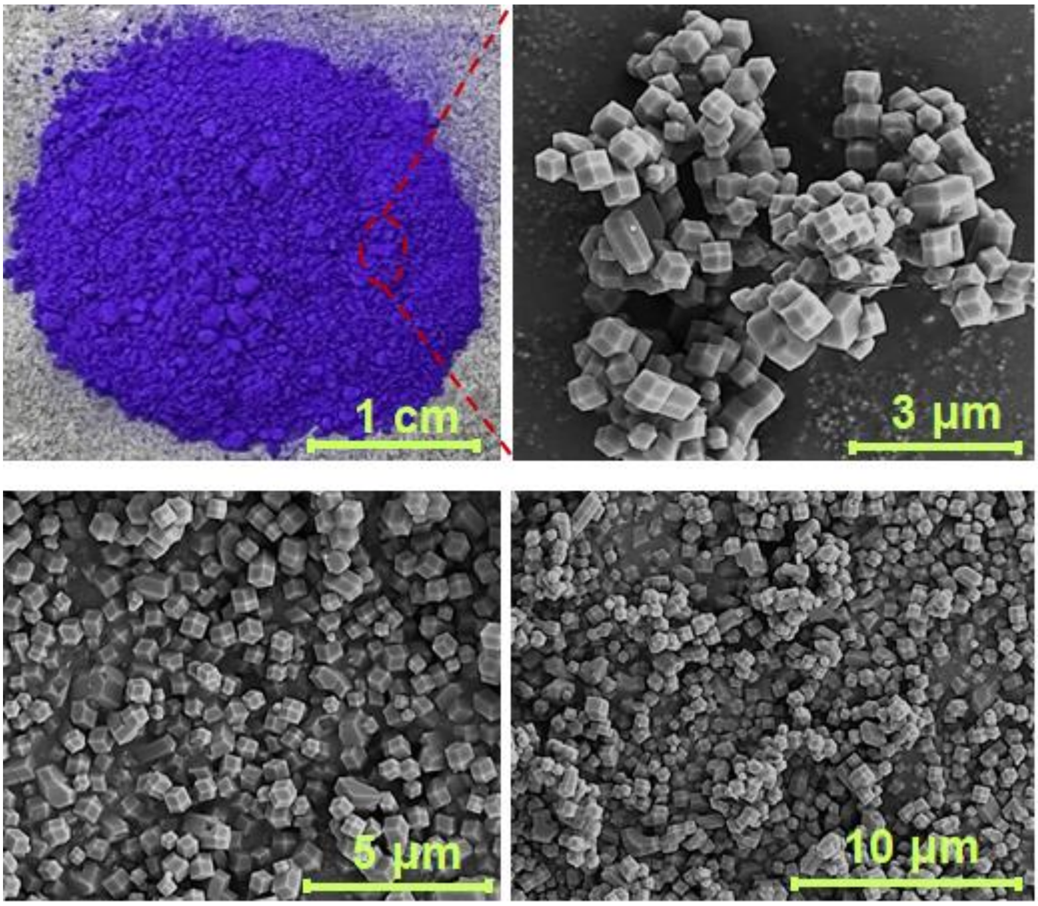

Supplementary Figure 9. ZIF-67 powder and its SEM images at different magnifications.

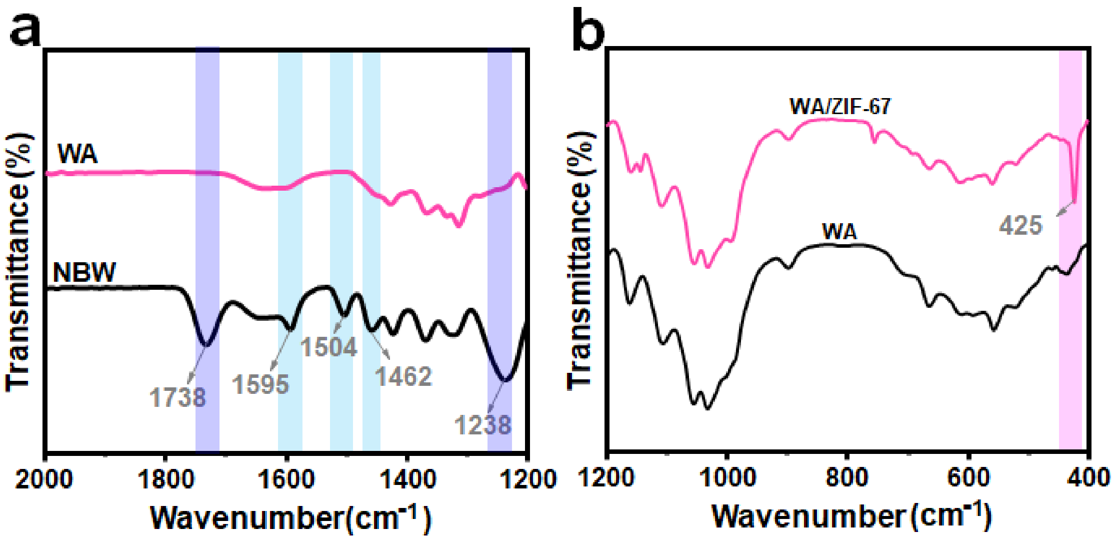

Supplementary Figure 10. FT-IR spectra of (a) NBW and WA at a wavenumber of 2000-1200 cm^{-1}, (b) WA and WA/ZIF-67 at a wavenumber of 1200-400 cm^{-1}.

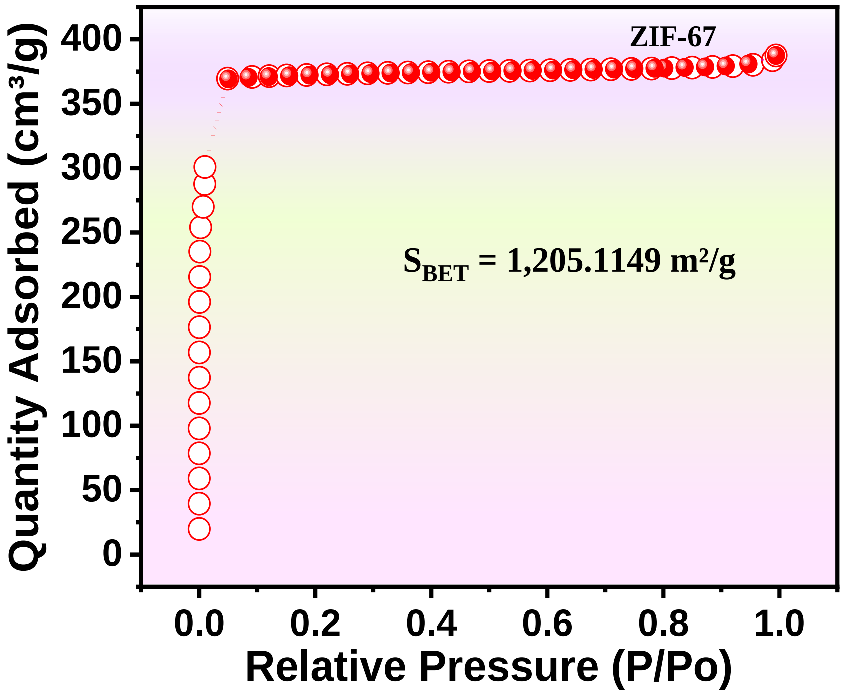

Supplementary Figure 11. Nitrogen adsorption desorption isotherm of ZIF-67.

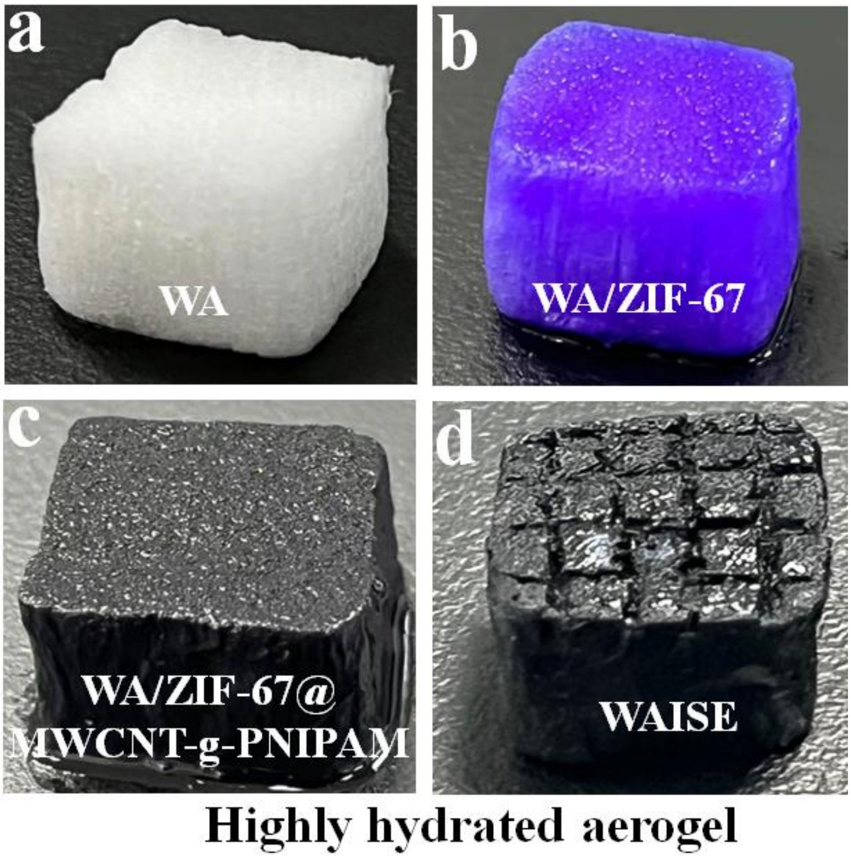

Supplementary Figure 12. Hydration state of wood aerogel materials by water absorption. (a) WA. (b) WA/ZIF-67. (c) WA/ZIF-67@MWCNT-g-PNIPAM. (d) WAISE.

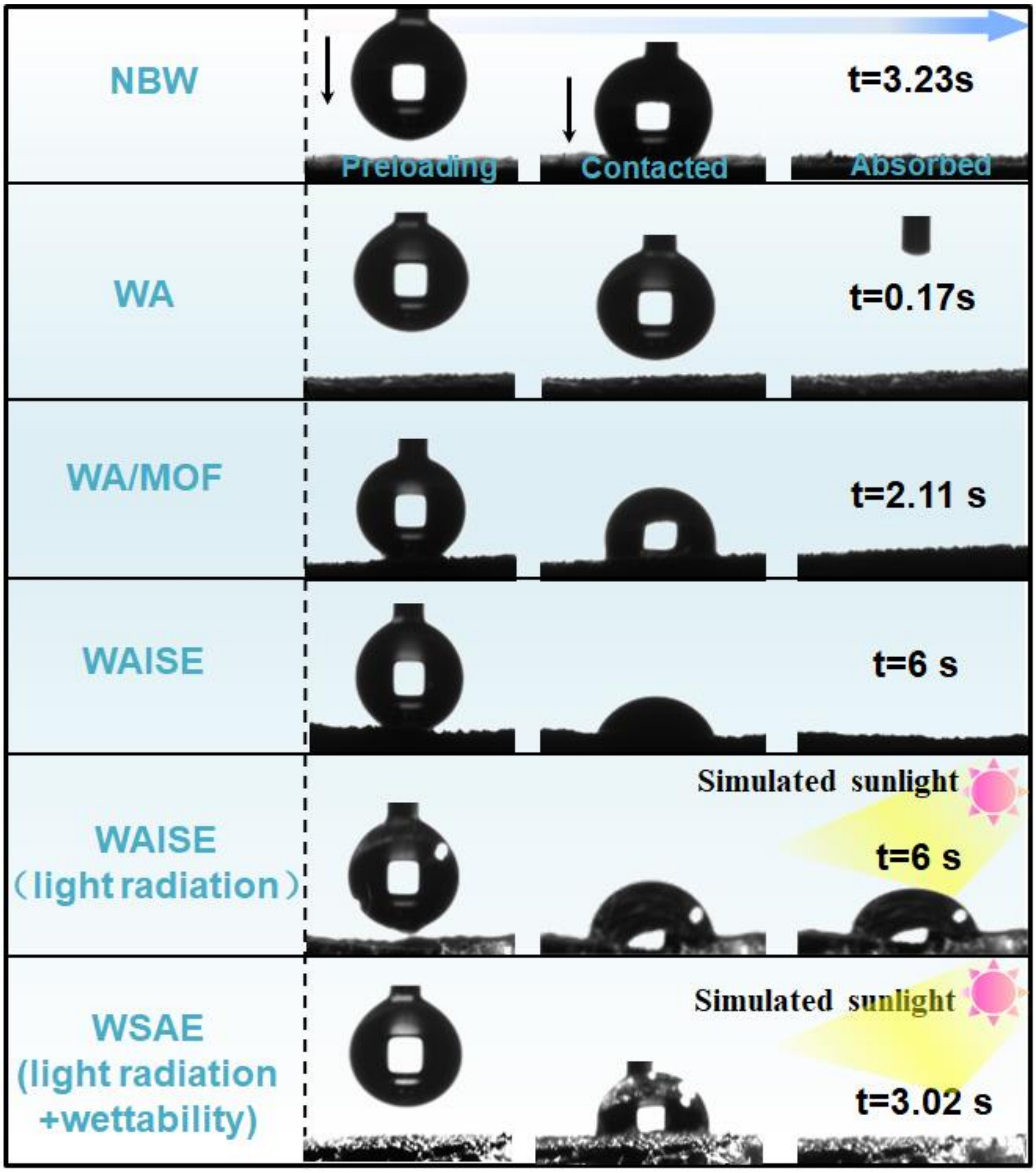

Supplementary Figure 13. The wettability was tested of NBW, WA, WA/ZIF-67, WAISE (under different conditions).

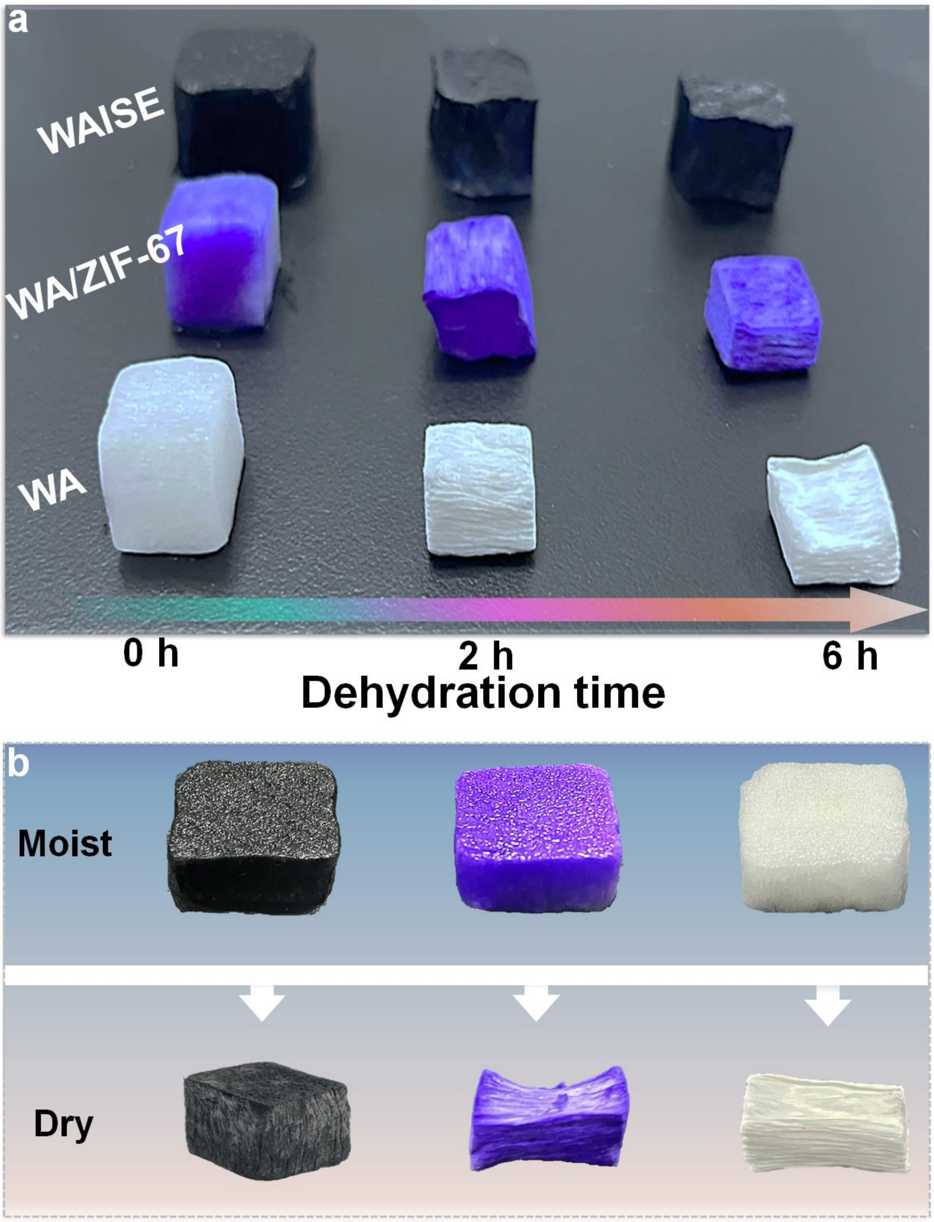

Supplementary Figure 14. (a) Optical photogram of the dehydration process at 0, 2, 6h (50 °C in oven environment). (b) High resolution optical photographs before and after drying.

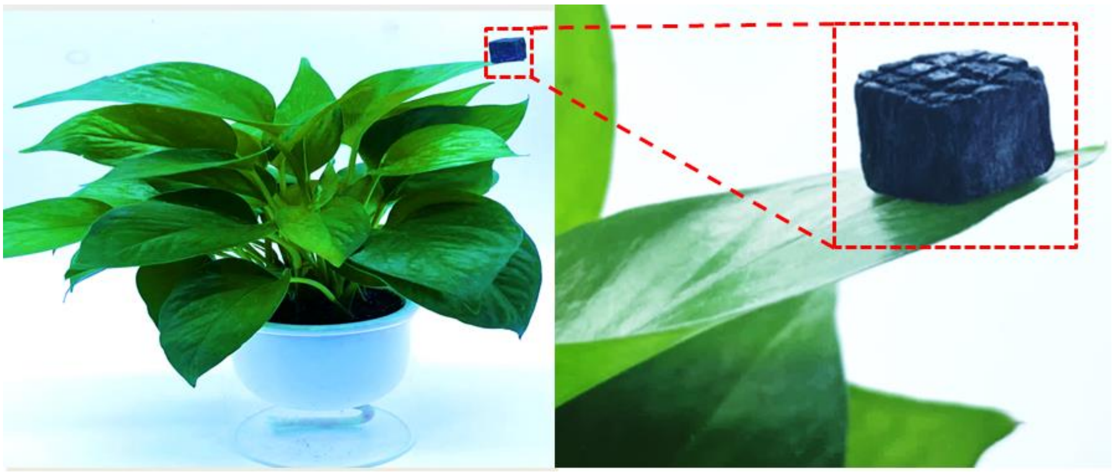

Supplementary Figure 15. Optical photo of WAISE placed on the end of a green plant leaf.

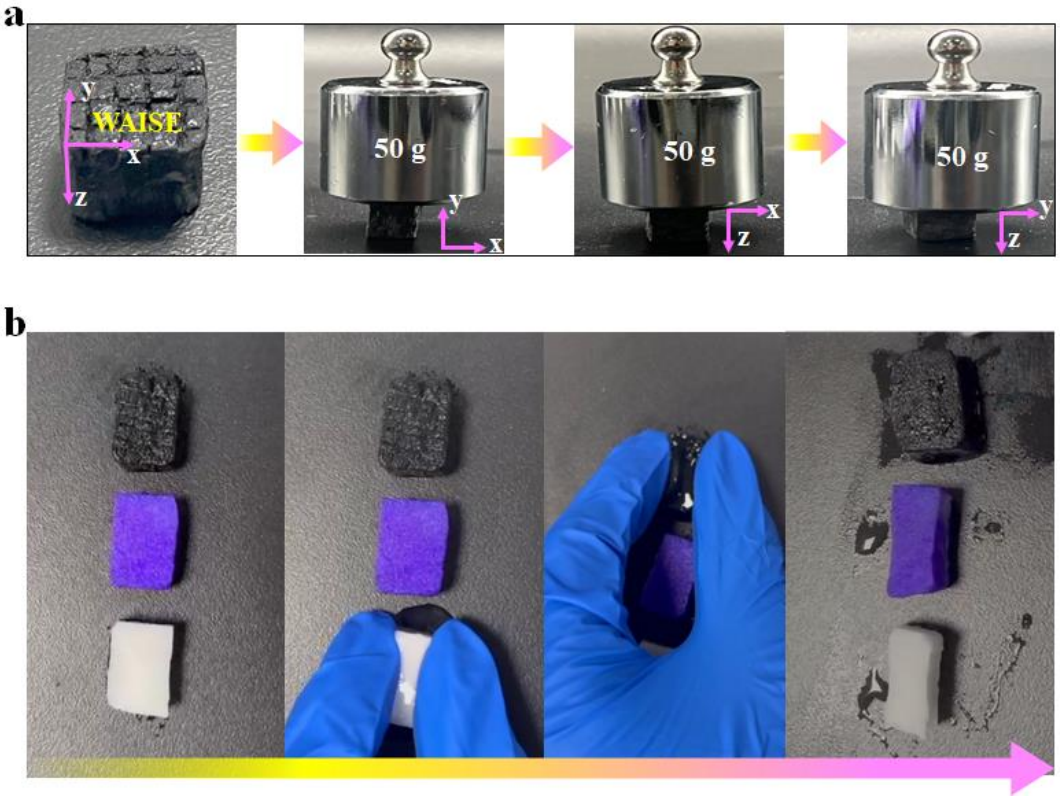

Supplementary Figure 16. (a) Load-bearing test was performed of WAISE in different directions. (b) The water-saturated material was squeezed to observe whether the shape could be restored.

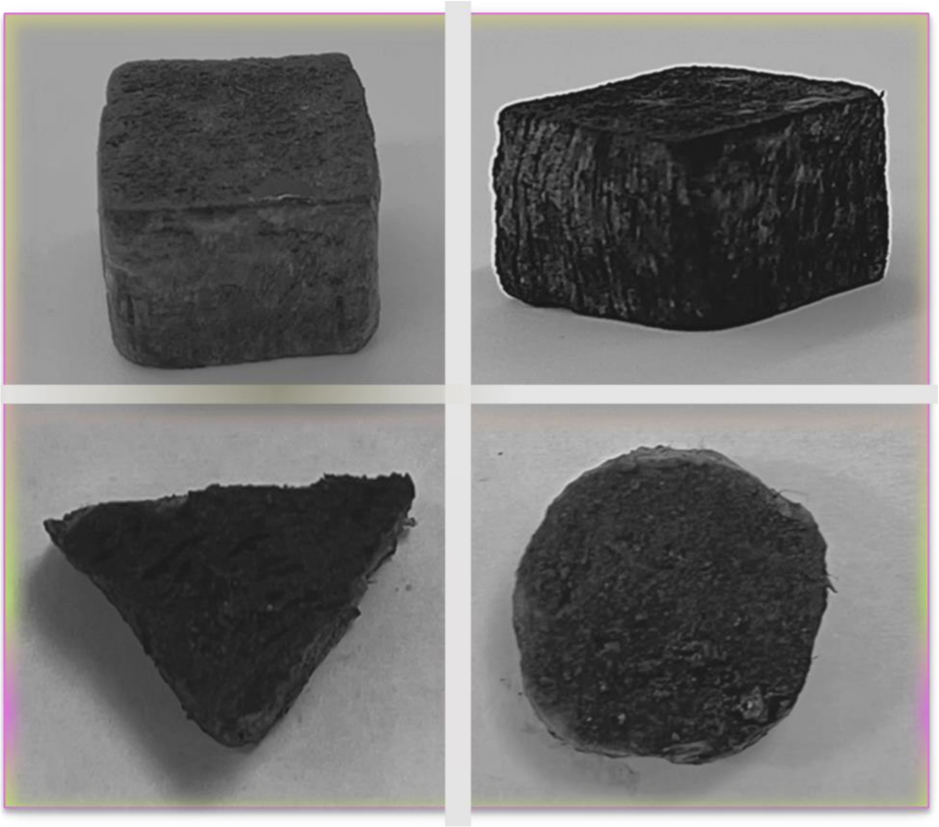

Supplementary Figure 17. Different shapes of evaporator materials.

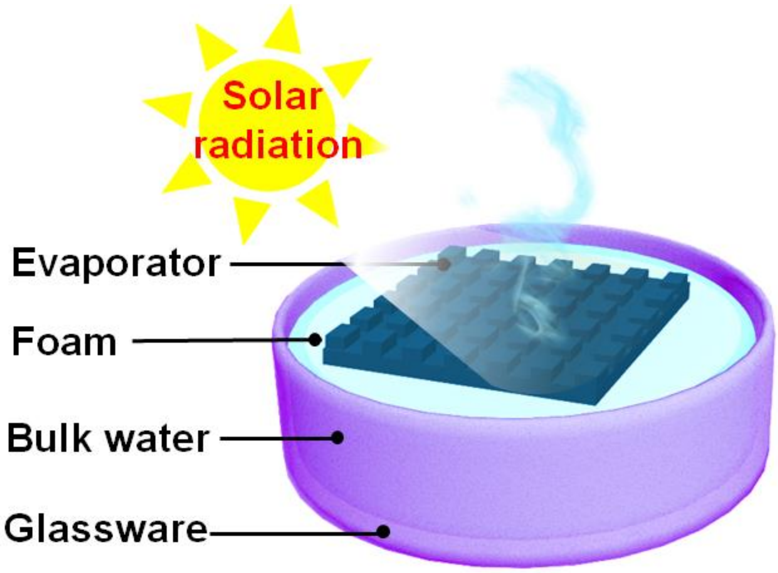

Supplementary Figure 18. Schematic diagram of the structure of the evaporation unit.

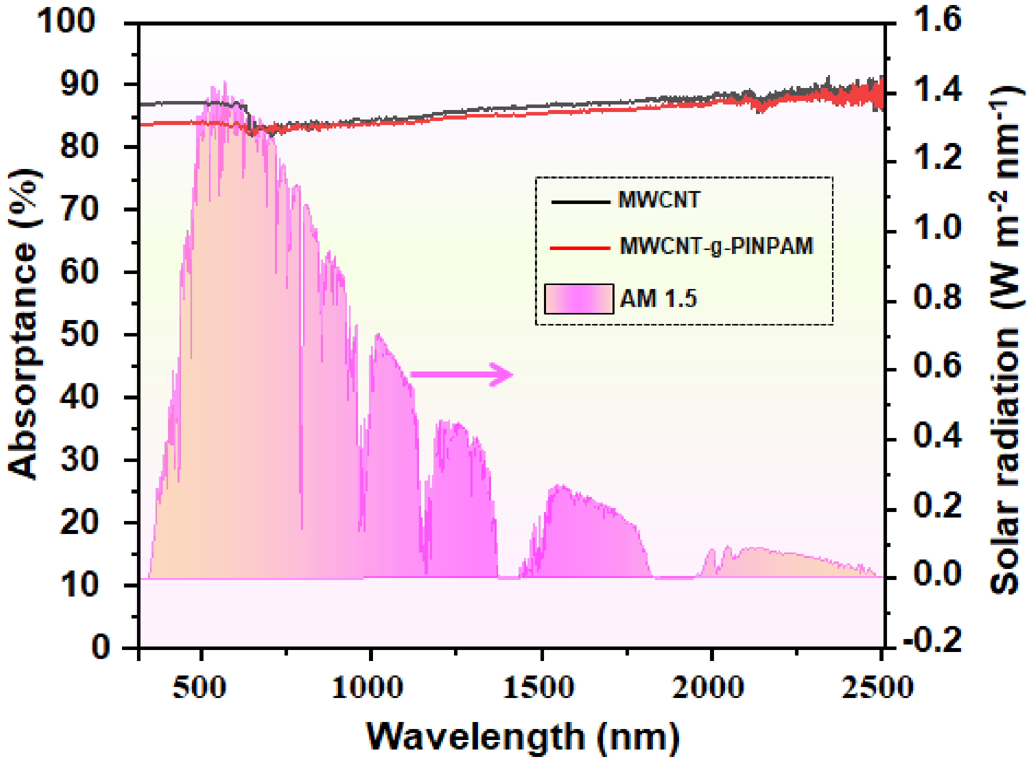

Supplementary Figure 19. Light absorption spectra of MWCNT and MWCNT-g-PNIPAM.

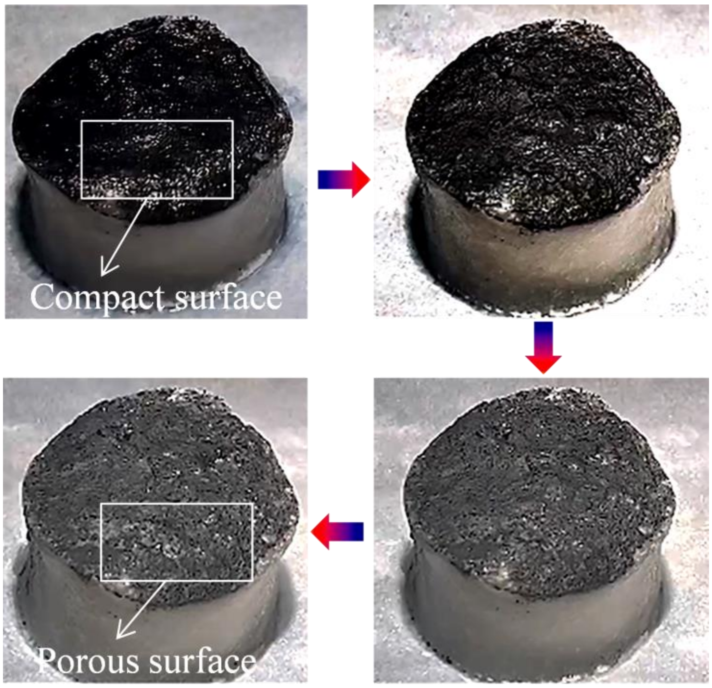

Supplementary Figure 20. Surface structural changes of MWCNT-g-PNIPAM coatings on soft cellulose aerogel surfaces with light exposure time.

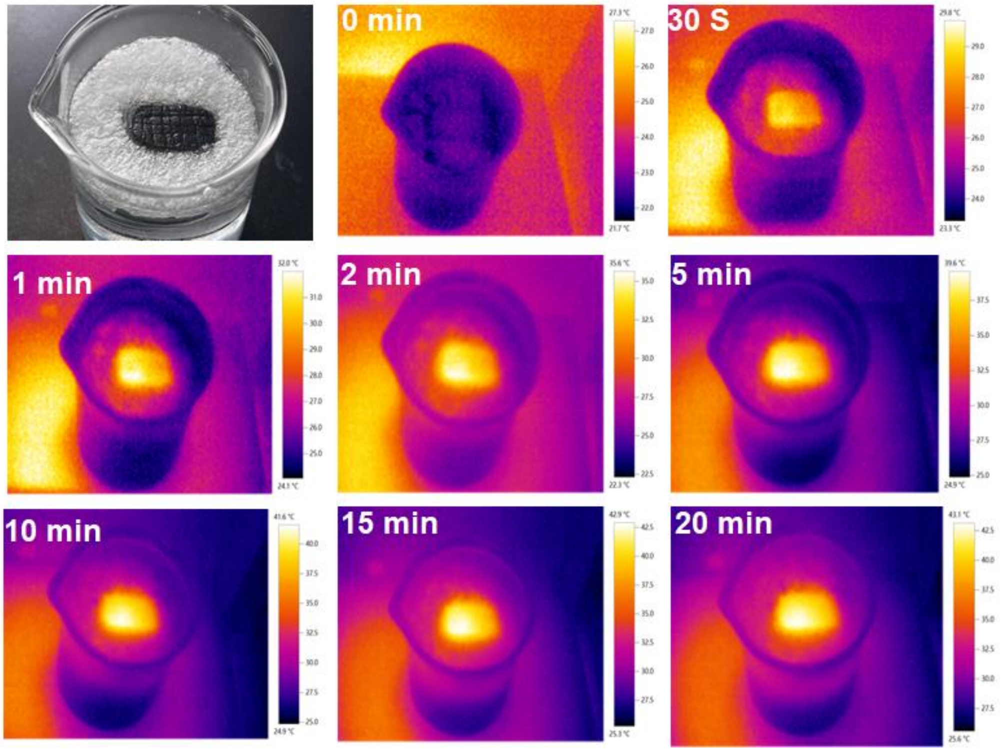

Supplementary Figure 21. Infrared thermography images of WAISE with foam protection in 20 min.

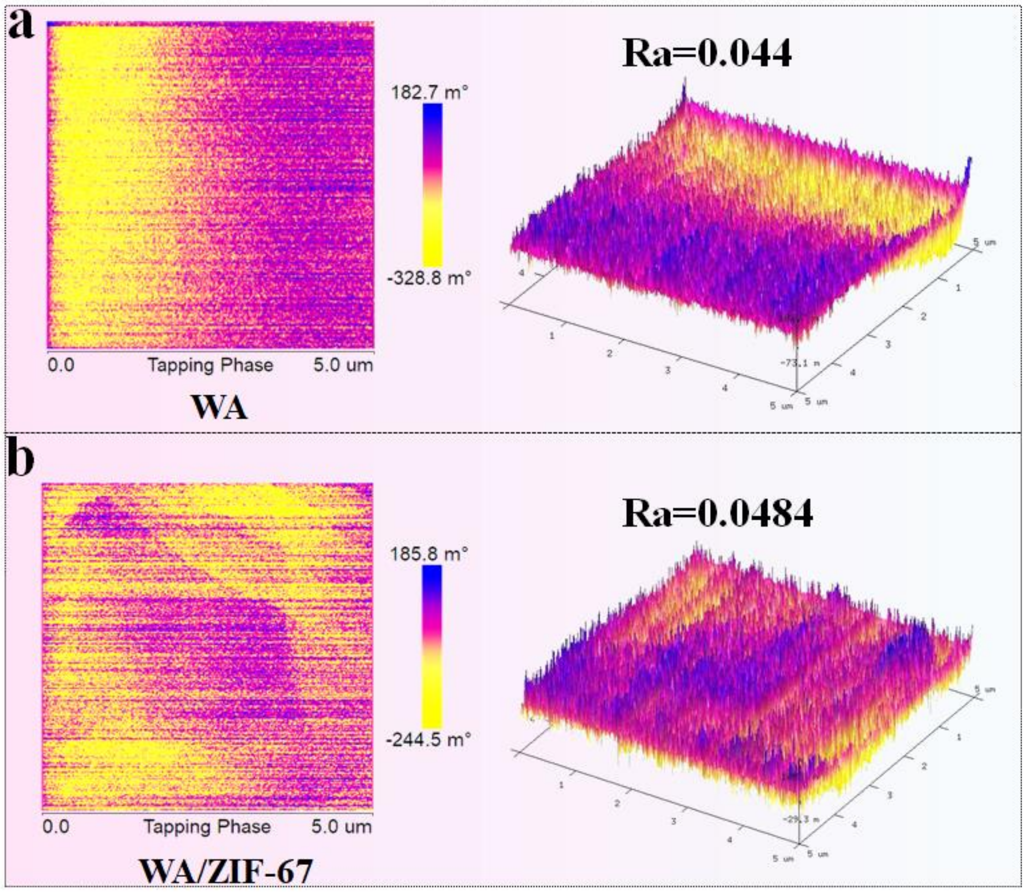

Supplementary Figure 22. The roughness was tested by AFM. (a) AFM images of WA. (b) AFM images of WA/ZIF-67.

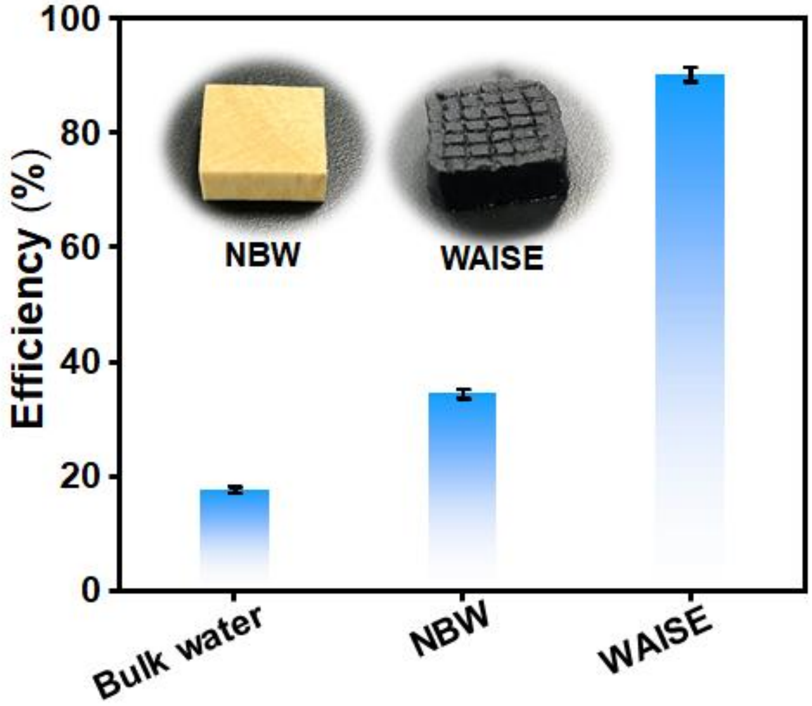

Supplementary Figure 23. Comparison of evaporation efficiency of different materials under 1 sun.

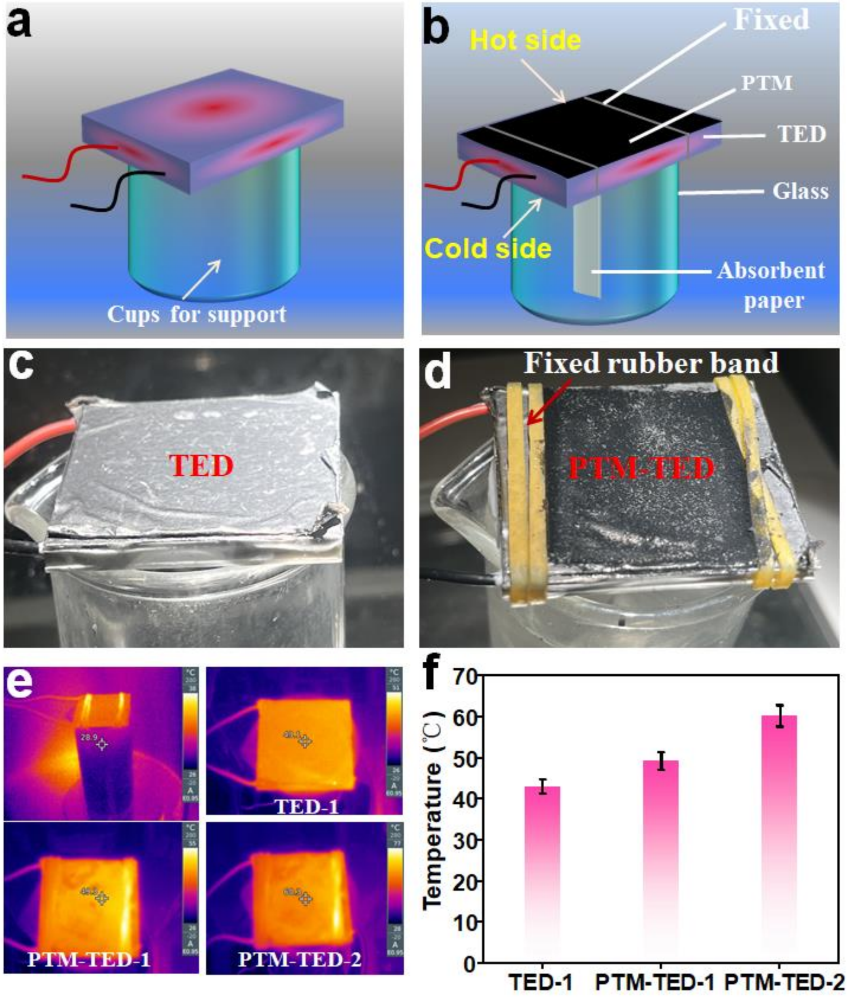

Supplementary Figure 24. Structural and thermal performance tests of TED and PTM-TED. (a) structure of TED. (b) structure of PTM-TED. (c) digital photographs of TED. (d) digital photographs of PTM-TED. (e) Infrared thermal imaging. (f) Maximum surface temperature.

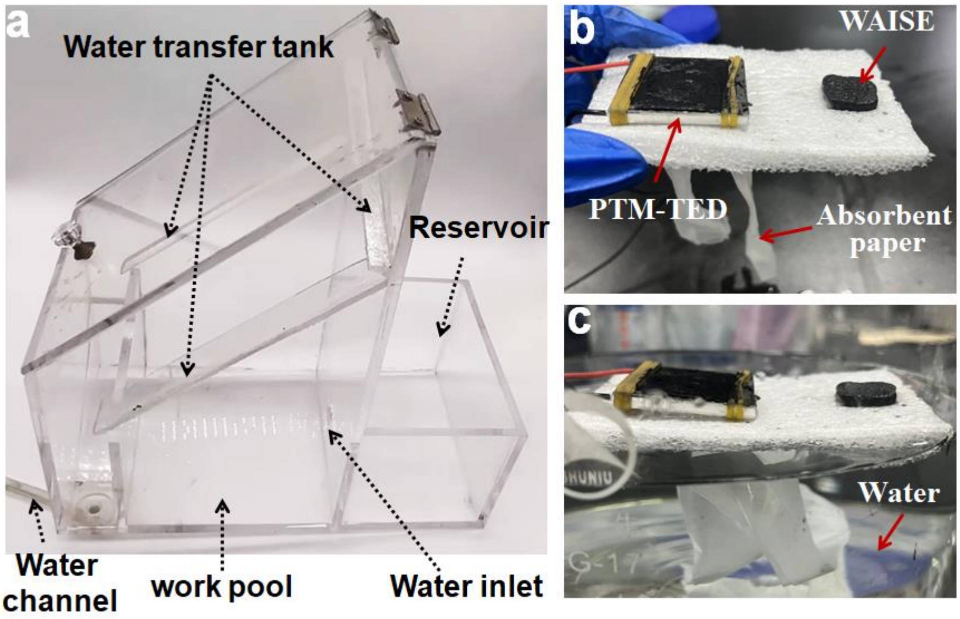

Supplementary Figure 25. Schematic diagram of the composition and structure of EGIS. (a) Water harvesting devices. (b) Evaporation-generation units. (c) Evaporation-generation units floating on the water surface.

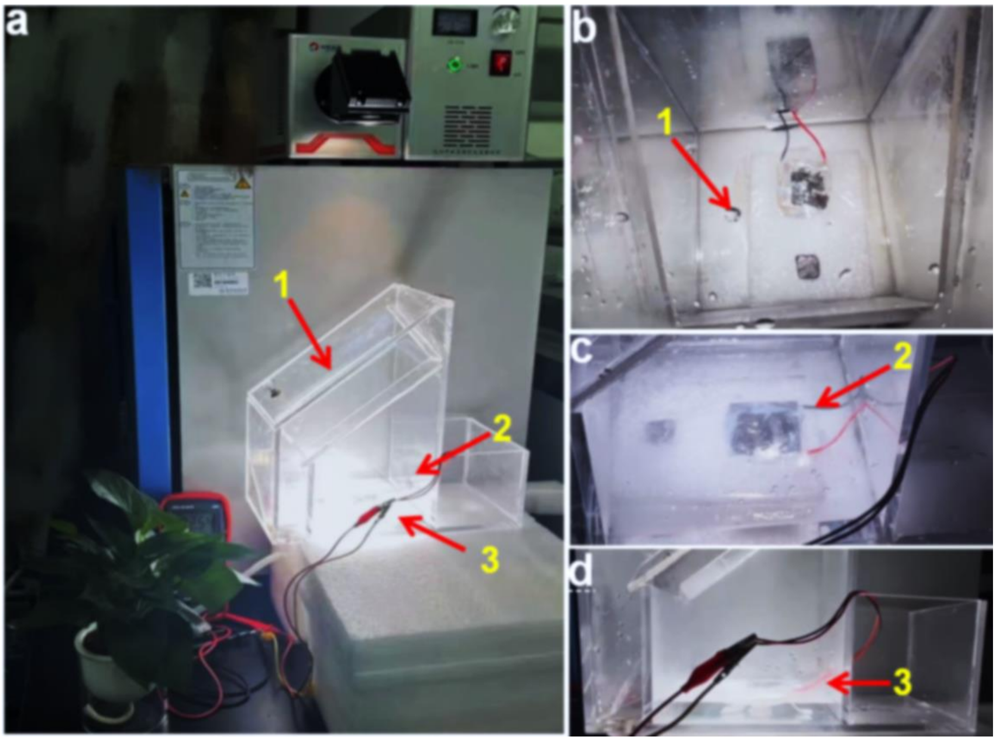

Supplementary Figure 26. The evaporation-generation-irrigation system (EGIS) was observed of from different viewpoints. (a) full view. (b) viewpoint 1. (c) viewpoint 2. (d) viewpoint 3.

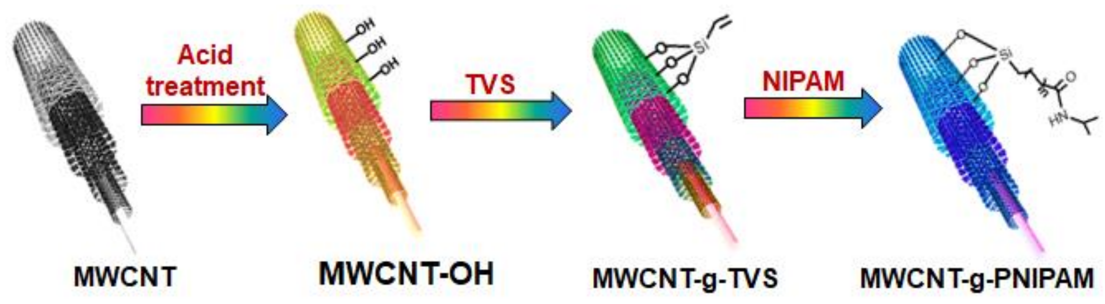

Supplementary Figure 27. Synthetic of MWCNT-g-PNIPAM.

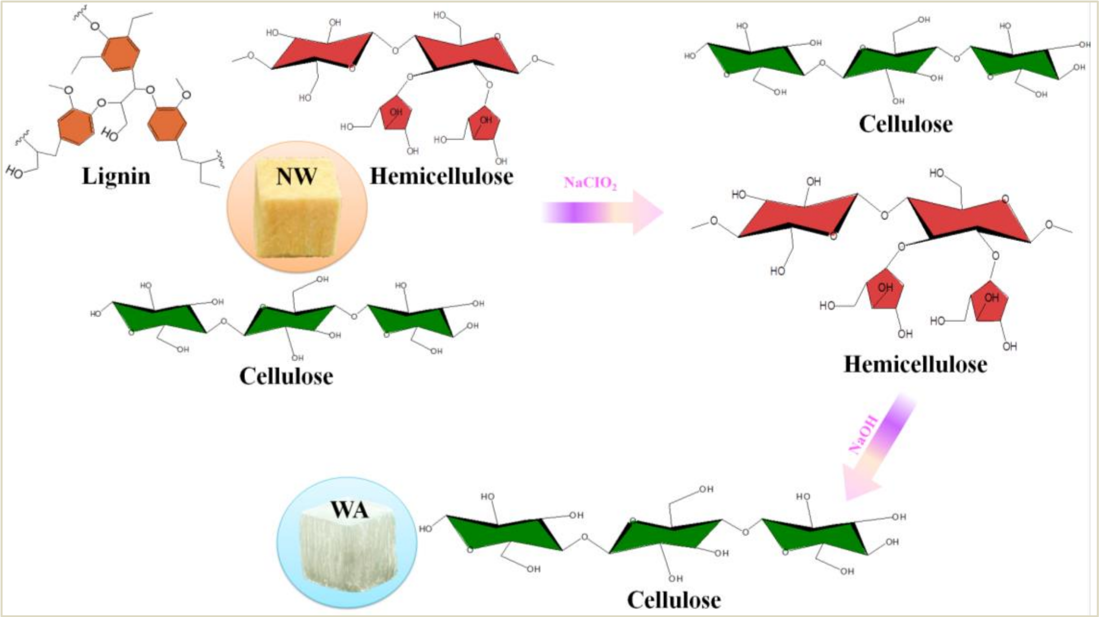

Supplementary Figure 28. The lignin and hemicellulose of NW was removed to obtain WA (the main component was cellulose).

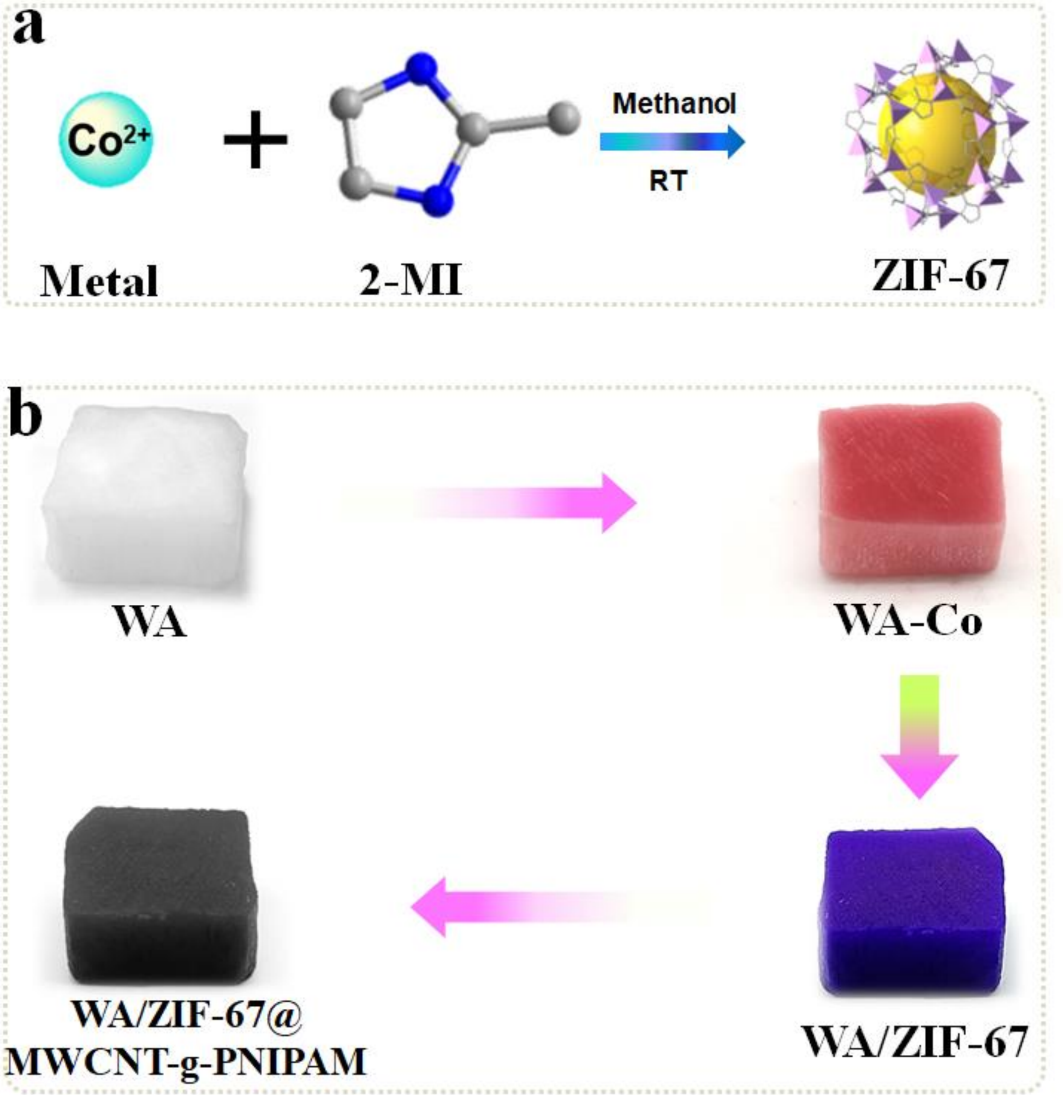

Supplementary Figure 29. (a) Synthesis mechanism diagram of ZIF-67 (b) Changes in macroscopic morphology of the WA/ZIF-67@MWCNT-g-PNIPAM evaporator preparation process.

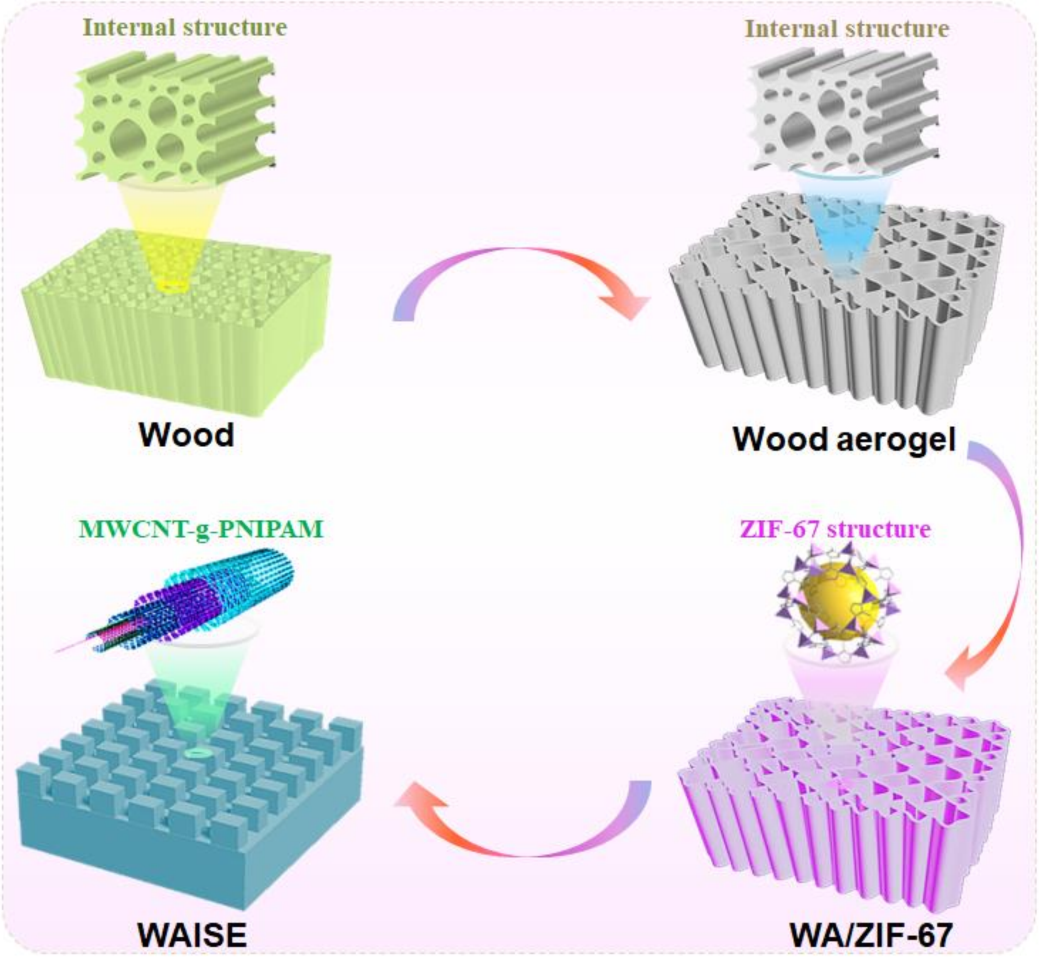

Supplementary Figure 30. Schematic diagram of the composition change of WA surface during evaporator prepare process.

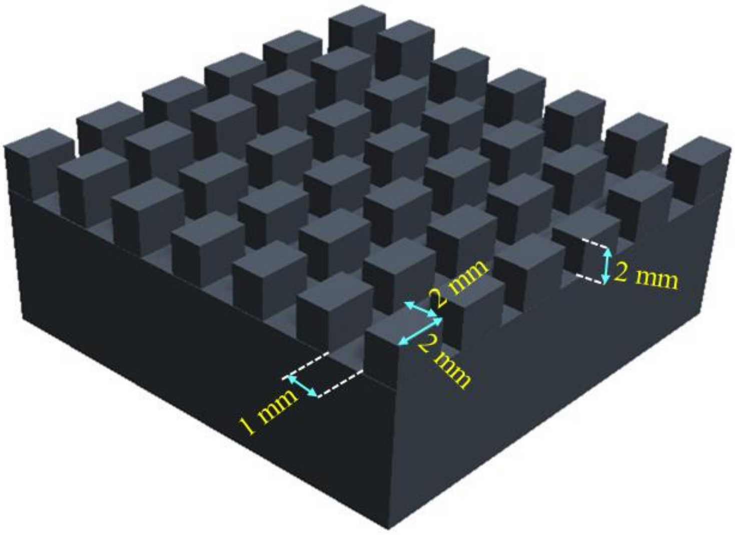

Supplementary Figure 31. Schematic diagram of the composition change of WA surface pores during evaporator preparation.

S4. Supplementary Table

Supplementary Table 1. Comparison table of evaporation performance under 1 sun

Name of evaporator	Salinity	Efficiency	Evaporation rate	References
Carbonized with structure	20	75.1	1.46	[S2]
PPy decorated wood	3.5	72.5	1.02	[S3]
An hierarchical solar steam generator on bacterial cellulose	3.5	80	2.9	[S4]
A surface-carbonized longitudinal wood membrane	3.5	83.6	2.43	[S5]
Wood-Based ZIF-8@PDA Functional Composites	3.5	86	2.7	[S6]
Janus wood	20	82	1.2	[S7]
HEA-nanoparticles-balsawood	20	-	2.58	[S8]
This work	20	86.5	3.34	-

S5. Supplementary Movie

Supplementary Movie 1:

WA/water and WA/ZIF-67/water units were established, and the dynamic distribution of water molecules in the two units was simulated (Simulate the evaporation process video using twice the playback speed).

Supplementary Movie 2:

The evaporation process Water and MWCNT-g-PNIPAM/water units were simulated (Simulate the evaporation process video using twice the playback speed).

S6. Supplementary References

[S1]. H. G. Geng, A facile approach to light weight, high porosity cellulose aerogels. *Int. J. Biol. Macromol*. **2018**, *118*, 921-931.

[S2]. Y. Kuang, C. Chen, S. He, E. M. Hitz, Y. Wang, W. Gan, R. Mi and L. Hu, *Adv. Mater*. **2019**, *31*, 1900498.

[S3]. Z. Wang, Y. Yan, X. Shen, C. Jin, Q. Sun and H. Li, *J. Mater. Chem. A*. **2019**, *7*, 20706-20712.

[S4]. Q.-F. Guan, Z.-M. Han, Z.-C. Ling, H.-B. Yang and S.-H. Yu, *Nano Lett.*, **2020**, *20*, 5699-5704.

[S5]. H. Liu, R. Jin, S. Duan, Y. Ju, Z. Wang, K. Yang, B. Wang, B. Wang, Y. Yao and F. Chen, *Small*, **2021**, *17*, 2100969.

[S6]. Y. Lu, D. Fan, Z. Shen, H. Zhang, H. Xu and X. Yang, *Nano Energy* **2022**, *95*, 107016.

[S7]. X. Chen, S. He, M. M. Falinski, Y. Wang, T. Li, S. Zheng, D. Sun, J. Dai, Y. Bian, X. Zhu, J. Jiang, L. Hu and Z. J. Ren, *Energy Environ. Sci.* **2021**, *14*, 5347-5357.

[S8]. Y. Li, Y. Ma, Y. Liao, L. Ji, R. Zhao, D. Zhu, X. Hu, G. Qin, H. Rong and X. Zhang, *Adv. Energy Mater.* **2022**, *12*, 2203057.
